# Rossellid glass sponges (Porifera, Hexactinellida) from New Zealand waters, with description of one new genus and six new species

**DOI:** 10.3897/zookeys.1060.63307

**Published:** 2021-09-17

**Authors:** Henry M. Reiswig, Martin Dohrmann, Michelle Kelly, Sadie Mills, Peter J. Schupp, Gert Wörheide

**Affiliations:** 1 Biology Department, University of Victoria, Victoria, British Columbia, Canada; 2 Department of Earth and Environmental Sciences, Palaeontology and Geobiology, Ludwig-Maximilians-Universität München, München, Germany; 3 Coasts and Oceans National Centre, National Institute of Water and Atmospheric Research, Auckland, New Zealand; 4 NIWA Invertebrate Collection, National Institute of Water and Atmospheric Research, Wellington, New Zealand; 5 ICBM Terramare, University of Oldenburg, Wilhelmshaven, Germany; 6 Helmholtz Institute for Functional Marine Biodiversity at the University of Oldenburg (HIFMB), Oldenburg, Germany; 7 SNSB – Bayerische Staatssammlung für Paläontologie und Geologie, München, Germany; 8 GeoBio-Center, Ludwig-Maximilians-Universität, München, Germany

**Keywords:** *
Bathydorus
*, *
Caulophacus
*, Hexasterophora, Lyssacinosida, *Nubes* gen. nov., ROV Kiel 6000, RV Sonne, *
Scyphidium
*

## Abstract

New Zealand’s surrounding deep waters have become known as a diversity hotspot for glass sponges (Porifera: Hexactinellida) in recent years, and description and collection efforts are continuing. Here we report on eight rossellids (Hexasterophora: Lyssacinosida: Rossellidae) collected during the 2017 RV Sonne cruise SO254 by ROV Kiel 6000 as part of Project PoribacNewZ of the University of Oldenburg, Germany. The material includes six species new to science, two of which are assigned to a so far undescribed genus; we further re-describe two previously known species. The known extant rossellid diversity from the New Zealand region is thus almost doubled, from nine species in five genera to 17 species in eight genera. The specimens described here are only a small fraction of hexactinellids collected on cruise SO254. Unfortunately, the first author passed away while working on this collection, only being able to complete the nine descriptions reported here. The paper concludes with an obituary to him, the world-leading expert on glass sponge taxonomy who will be greatly missed.

## Introduction

The deep sea of the New Zealand region has only recently been recognised as a hotspot of glass sponge diversity, with two major monographs treating the dictyonal and euplectellid hexactinellids (Reiswig and Kelly 2011, 2018). However, the family Rossellidae Schulze, 1885 (order Lyssacinosida Zittel, 1877), has not been extensively treated thus far. In a literature review of the sponge fauna of New Zealand, Dawson (1993) listed three species of rossellid glass sponges in New Zealand waters: *Aulochonecylindrica* Schulze, 1886 [now accepted as Crateromorpha (Aulochone) cylindrica], *Rossellaijimai* Dendy, 1924, and *Symplectellarowi* Dendy, 1924 (see also Dohrmann 2016). *Symplectellarowi*, and to a lesser extent, *Rossellaijimai*, are now fairly well known because they occur in relatively shallow water in many parts of the New Zealand Exclusive Economic Zone, and they have distinctive morphologies that are easily identifiable from images captured in situ by deep-water imaging systems.

The endemic genus *Symplectella* Dendy, 1924 and only known species, *S.rowi*, was first collected from the Terra Nova Expedition Station 96, 7 miles (11.5 km) east of North Cape on the eastern tip of the North Island, at a depth of 70 fathoms (128 m). It is now known to be relatively common around New Zealand, from the type locality south along the East Coast to the Bay of Plenty, East Cape. In the South Island, the distribution extends from southeast of Cook Strait and Kaikoura Coast out onto the Chatham Rise and deep into the subantarctic New Zealand region. *Symplectellarowi* is less common on the West Coast of both Islands, but this is not an unusual distribution pattern for New Zealand Porifera and may reflect the lighter collection effort on that coastline. The species is, however, quite common in Fiordland (Battershill et al. 2010), where it is found in deep SCUBA-diving depths (up to ~ 30 m), along with other attractions such as endemic black and red corals, hydrozoan sea fans, and sea pens that also occur in shallow depths and are impressive tourist attractions. Recently, several important regional populations have come to light in the North Island: off Rakitu Island, Great Barrier Island, Hauraki Gulf (Lee et al. 2015; Kelly 2016); off Mimiwhangata, Northland (Kerr and Doak, pers. comm.); and in the North Taranaki Bight (Jones et al. 2018).

*Rossellaijimai* was collected from the same Terra Nova Expedition station as *S.rowi*, ~ 12 km east of North Cape, at 128 m. It is now known from the continental shelf around Northland on both the west and east coasts, and on the Chatham Rise. *Rossellaijimai* and *S.rowi* often co-occur and so the important North Island regional populations of the more abundant *S.rowi* include the odd specimen of *R.ijimai*. Of special interest is the North Taranaki Bight population, discovered only in 2018, where the two species co-occur in relatively high numbers. Recent NIWA and Department of Conservation ROV surveys around Fiordland revealed the first, albeit unconfirmed record of *R.ijimai* (Page and Handley, pers. comm.).

In the 2009 inventory of New Zealand biodiversity, Kelly et al. (2009) listed the following species, confirmed in a later draft manuscript on Rossellidae under preparation by HMR and MK. These include *Caulophacushadalis* Lévi, 1964, now accepted as Caulophacus (Caulophacus) hadalis (not included in Dawson 1993), *Crateromorphacylindrica* (Schulze, 1886), now accepted as Crateromorpha (Aulochone) cylindrica, and *Caulophacusschulzei* Wilson, 1904, now accepted as Caulophacus (C.) schulzei. Several species were also included and confirmed for New Zealand in Kelly et al. (2009), from a draft 1980 manuscript under preparation by HMR: Crateromorpha (Aulochone) haliprum Tabachnick & Lévi, 2004; Crateromorpha (Caledochone) caledoniensis Tabachnick & Lévi in Tabachnick (2002); Caulophacus (Caulodiscus) onychohexactinus Tabachnick & Lévi, 2004; *Sympagellaclavipinula* Tabachnick & Lévi, 2004. These species are beyond the scope of this work and will be dealt with later. Kelly et al. (2015) confirmed the presence of *Caulophacus (C.) hadalis and Crateromorpha* (*A.*) *cylindrica* in their Kermadec Islands review.

In 2013, several well-preserved body fossils of a new species, *Rossellacylindrica* Buckeridge & Kelly, 2013 (in Buckeridge et al. 2013), from the late Palaeocene-early Eocene Red Bluff Tuff of Chatham Island, were confirmed by HMR. Both *R.antarctica* Carter, 1872 and *R.racovitzae* Topsent, 1901 were stated to be present on the Chatham Rise, but this remains unconfirmed. Various Rossellidae species were also represented in the Oamaru Diatomite (Eocene) as hexactins, pentactins, and stauractins, illustrated in Hinde and Holmes (1892), but microfossil spicules were not recorded in the Tutuiri Greensand (Kelly and Buckeridge 2005), despite the relatively high proportion of hexactinellid taxa in the fauna. Specimens compared to *Rossellaracovitzae/R*. *antarctica* on the Chatham Rise by Buckeridge et al. (2013) and Campbell Plateau (Chin and Kelly, pers. comm.) form a small, squat, open-mouthed barrel with a restricted base, and have a characteristic veil of hypodermal pentactins protruding beyond the surface of the sponge wall. This latter character is highly reminiscent of the new species described herein, *Nubestubulata* gen. nov., sp. nov. and *Scyphidiumvariospinosum* sp. nov., the type localities of which are just north of Chatham Rise.

The 2017 German RV Sonne (cruise SO254) expedition to New Zealand afforded an important collection of ~ 100 new hexactinellid specimens and corresponding seafloor images, collected as part of Project PoribacNewZ of the Institute for Chemistry and Biology of the Marine Environment (ICBM), Carl von Ossietzky University of Oldenburg, using the GEOMAR Helmholtz Centre for Ocean Research Kiel Remotely Operated Vehicle (ROV) Kiel 6000 (Schupp et al. 2017). Preparation of a manuscript combining morphological descriptions and molecular systematics of these glass sponges was underway when we were devastated by the untimely death of Henry Reiswig in July 2020. The objective of this work is to provide the descriptions of specimens completed prior to Henry’s passing. This work includes the establishment of a new endemic genus, *Nubes* gen. nov., with two new species; a new species of *Bathydorus* Schulze, 1886; redescription of *Scyphidiumaustraliense* Tabachnick, Janussen & Menschenina, 2008, and description of a new species of *Scyphidium* Schulze, 1900; redescription of Caulophacus (Caulophacus) discohexaster Tabachnick & Lévi, 2004, and description of two new species of Caulophacus (Caulophacus) Schulze, 1886.

## Materials and methods

### Sample collection

**Figure 1.**
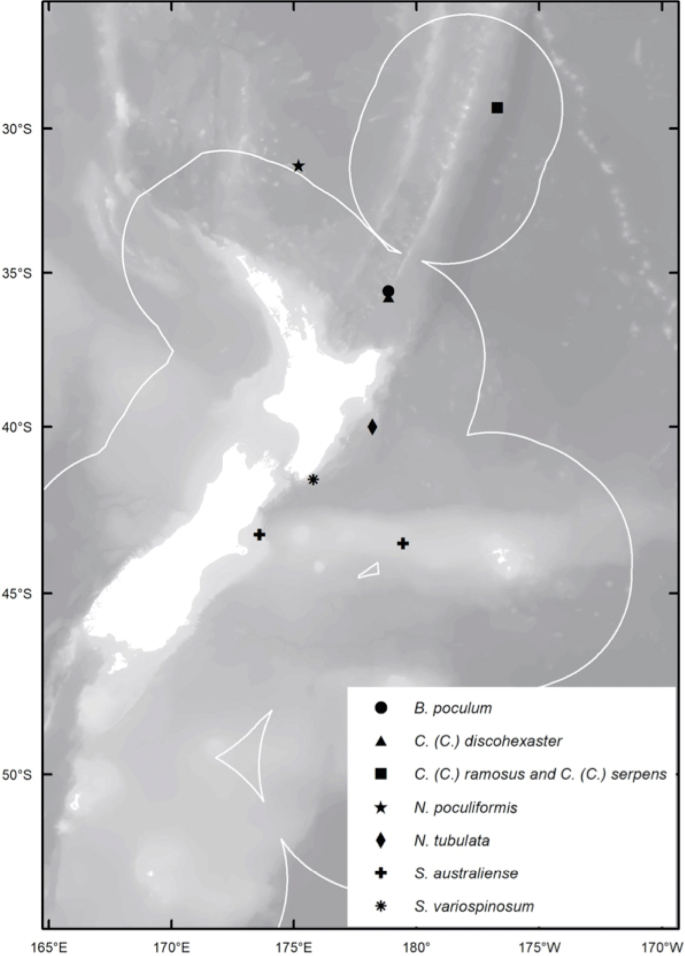
Study area showing the distribution of newly described rossellid sponges in the New Zealand EEZ and in international waters.

Specimens, seafloor images, and videos were collected as part of Project PoribacNewZ of the Institute for Chemistry and Biology of the Marine Environment (**ICBM**), Carl von Ossietzky University of Oldenburg, on the new German RV Sonne (cruise SO254), using the GEOMAR Helmholtz Centre for Ocean Research Kiel ROV Kiel 6000 (Schupp et al. 2017). With the exception of NIWA 126016, which was collected in International Waters to the east of Norfolk Island and the Three Kings Ridge, all other specimens were collected from the New Zealand Exclusive Economic Zone (**EEZ**); collection sites and general distribution of the species are shown in Fig. 1.

### Sample preparation

Subsamples were taken on board, stored in appropriate preservatives for morphological and molecular work, and shipped to the Ludwig-Maximilians-Universität (**LMU**) Munich. Specimens reported here, together with a much larger collection, the remainder of which will be reported on elsewhere, were first subjected to a molecular phylogenetic study (results not shown) based on a mitochondrial 16S ribosomal DNA fragment (cf. Dohrmann et al. 2008) for initial assessment of their relationships. This then allowed selection of interesting specimens for further study, some of which we describe below. Preliminary morphological identifications of these specimens were made by MD by analysing spicule content with light microscopy of temporary bleach digestions of tissue pieces. Sample preparation for formal identification and description of taxa new to science (as well as re-descriptions of known species where appropriate) were then performed by HMR using methods described in Reiswig and Kelly (2011, 2018).

### Registration of type and general material

Primary and secondary type materials of new species and additional material are deposited in the Invertebrate Collection (**NIC**) at the National Institute of Water and Atmospheric Research (**NIWA**), Greta Point, Wellington, using the prefix NIWA – . Registration numbers are cited in the text. Taxonomic authority is restricted to Reiswig, Dohrmann & Kelly.

### Abbreviations

**EEZ** Exclusive Economic Zone;

**GEOMAR** Research Center for Marine Geosciences, Helmholtz Centre for Ocean Research Kiel, Germany;

**ICBM** Institute for Chemistry and Biology of the Marine Environment, Carl von Ossietzky University of Oldenburg;

**LM** light microscopy;

**NIC–NIWA** Invertebrate Collection, NIWA, Wellington, New Zealand;

**NIWA**National Institute of Water and Atmospheric Research, Wellington, New Zealand;

**SEM** scanning electron microscopy.

## Systematics

### PORIFERA Grant, 1836

**HEXACTINELLIDA Schmidt, 1870**

**HEXASTEROPHORA Schulze, 1886**

**LYSSACINOSIDA Zittel, 1877**

**ROSSELLIDAE Schulze, 1885**

#### ROSSELLINAE Schulze, 1885

##### Bathydorus

Taxon classificationAnimaliaLyssacinosidaRossellidae

Schulze, 1886

BD2A510B-B9AE-571B-90D9-D09ED0009107

Bathydorus poculum Reiswig, Dohrmann & Kelly, sp. nov.

##### Nubes

Taxon classificationAnimaliaLyssacinosidaRossellidae

Reiswig, Dohrmann & Kelly gen. nov.

6FC6D34E-962A-5A07-B310-E30EF356C4B2

Nubes tubulata Reiswig, Dohrmann & Kelly, sp. nov.
Nubes poculiformis Reiswig, Dohrmann & Kelly, sp. nov.

##### Scyphidium

Taxon classificationAnimaliaLyssacinosidaRossellidae

Schulze, 1900

EB3D7FF2-AE47-592E-A053-64008B0FAA8F

Scyphidium australiense Tabachnick, Janussen & Menschenina, 2008
Scyphidium variospinosum Reiswig, Dohrmann & Kelly, sp. nov.

#### LANUGINELLINAE Gray, 1872

##### Caulophacus (Caulophacus)

Taxon classificationAnimaliaLyssacinosidaRossellidae

Schulze, 1886

AAE57410-1859-5553-BD91-FCC23ABA0404

Caulophacus (Caulophacus) discohexaster Tabachnick & Lévi, 2004
Caulophacus (Caulophacus) serpens Reiswig, Dohrmann & Kelly, sp. nov.
Caulophacus (Caulophacus) ramosus Reiswig, Dohrmann & Kelly, sp. nov.

##### Rossellidae

Taxon classificationAnimaliaLyssacinosidaRossellidae

Schulze, 1885

2804D832-96EF-5122-B2E3-1DAB054E9B70

###### Diagnosis.

The body is usually cup-like basiphytose or lophophytose; in the pedunculate forms the body can be mushroom-like. Prostalia lateralia, when present, are formed with diactins or outwardly protruding hypodermal pentactins; prostalia basalia, when present, are outwardly protruding hypodermal pentactins which are usually specialised (anchorate). Choanosomal skeleton consists of diactins, sometimes together with less frequent hexactins. Hypodermal pentactins often present, usually they protrude from the dermal surface serving as prostalia. Hypoatrial pentactins are rarely found or absent in some taxa. Dermalia are combinations of various spicules usually pentactins; stauractins and diactins, rarely hexactins. Atrialia are usually hexactins but other holactinoidal spicules can be also found there. Microscleres are various: holactinoidal, asterous and asters; they usually have discoidal or oxyoidal terminations but sometimes floricoidal, onychoidal, or sigmoidal ones (after Tabachnick 2002).

##### Rossellinae

Taxon classificationAnimaliaLyssacinosidaRossellidae

Schulze, 1885

38826F23-7EEE-54DF-BD5D-86AE99F25A88

###### Diagnosis.

As for family.

###### Remarks.

This subfamily is clearly not monophyletic (Dohrmann et al. 2017) and retained here for historical reasons only.

##### Bathydorus

Taxon classificationAnimaliaLyssacinosidaRossellidae

Schulze, 1886

E5F13B62-E7BB-5693-AF46-95A9C64109DE

###### Diagnosis.

Rossellinae with tubular, saccular, or plate-like gross morphology. Basiphytous or lophophytous, thin-walled. Dermalia are combinations of spicules from hexactins to diactins. Regular pentactins make up a hypodermal layer. Choanosomal skeleton composed of diactins, sometimes with hexactins. Atrialia are hexactins or stauractins. Microscleres are combinations of oxyoidal hexasters, hemihexasters, and hexactins; lacking pappocomes (from Kahn et al. 2013).

###### Type species.

*Bathydorusfimbriatus* Schulze, 1886

##### Bathydorus poculum

Taxon classificationAnimaliaLyssacinosidaRossellidae

Reiswig, Dohrmann & Kelly sp. nov.

50ABE2C9-0B9C-59B7-89E7-DB39ABF4C497

http://zoobank.org/1E8B4837-7A12-4A08-91B6-5A8E63CC79F2

**Figure 2.**
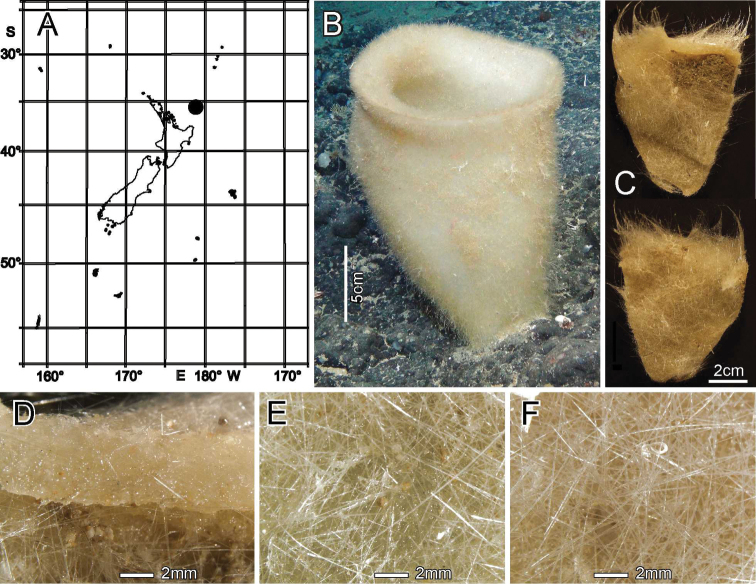
*Bathydoruspoculum* sp. nov., holotype NIWA 126338, distribution, skeleton, and morphology **A** distribution in New Zealand waters **B** holotype in situ (scale bar approximate) **C** dermal (upper) and atrial (lower) sides of the preserved main part of the collected fragment **D** magnified area of the oscular margin, showing the atrial surface curving out over the dermal surface **E** dermal surface with dense prostal diactins **F** atrial surface with similarly dense prostal diactins. Image **B** captured by ROV Team GEOMAR, ROV Kiel 6000 onboard RV Sonne (voyage SO254), courtesy of Project PoribacNewZ, GEOMAR, and ICBM.

**Figure 3.**
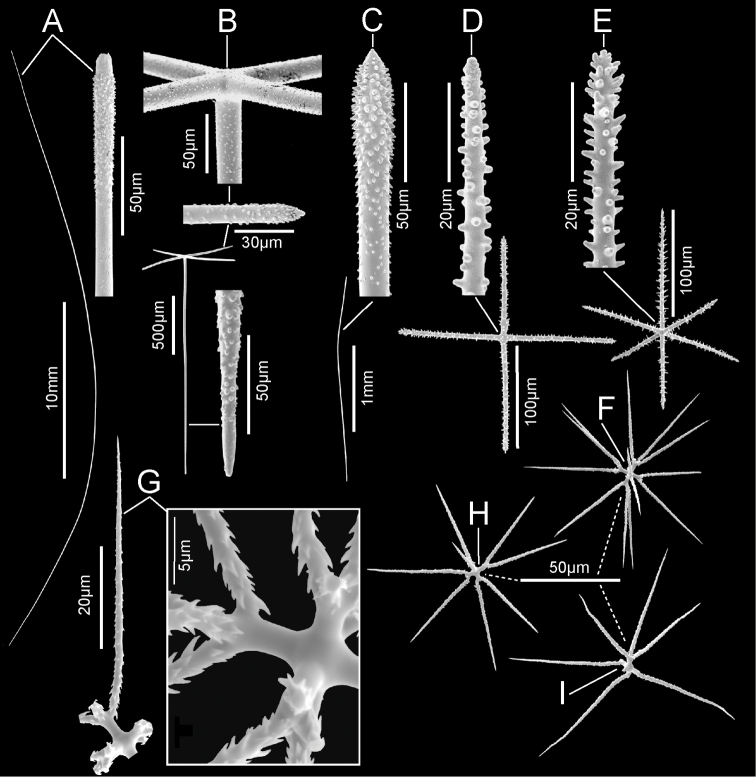
*Bathydoruspoculum* sp. nov., holotype NIWA 126338 spicules **A** prostal diactin, whole and enlarged end **B** hypodermal pentactin, whole and enlarged spicule centre, tangential and proximal ray ends **C** choanosomal diactin, whole and enlarged end **D** stauractine dermalium, whole and enlarged ray end **E** pinular hexactine atrialium, whole and enlarged ray end **F** oxyhexaster **G** enlarged whole primary and secondary ray (left) and centre of spicule showing smooth primary and ornamentation of spines on secondary rays (right) **H** hemioxyhexaster **I** hemioxystauraster.

Figs 2 , 3 ; Table 1

###### Material examined.

***Holotype***NIWA 126338, RV Sonne Stn SO254/85ROV19_BIOBOX17, Southern Kermadec Ridge, 35.612°S, 178.852°E, 1150 m, 24 Feb 2017.

###### Distribution.

Known only from the type locality, the Southern Kermadec Ridge, north of New Zealand (Fig. 2A).

###### Habitat.

Attached to hard substratum at 1149 m (Fig. 2B).

###### Description.

Morphology of the holotype is a thick-walled funnel attached to rock substratum by a wide basal disc (Fig. 2B). Both dermal and atrial surfaces have a very dense, bushy, cover of prostal diactins (Fig. 2C, E, F). The single terminal osculum is the widest body part and the margin is abruptly sharpened; it has no marginalia (Fig. 2D). Dimensions of the holotype are ~ 17.2 cm high and 12.8 cm wide; the measurements are only approximate as only one of the two laser points could be certainly found on the in-situ images. Wall thickness is 10.7 cm, excluding the 1–2.5 cm thick prostal cover layers on each side. Texture is soft, compressible, and resilient, neither hard nor fragile. Surfaces of both the inner and outer walls are hairy to the naked eye, and when inspected at low magnification of a dissecting microscope, both are covered with a bushy layer of prostal diactins. Colour in life is pale beige, and pale brown when preserved in ethanol.

***Skeleton*.** Choanosomal skeleton consists of a loose network of thin choanosomal diactins amongst the thicker proximal ends of prostal diactins, and proximal rays of hypodermal pentactins. No choanosomal hexactins are present. There is no evidence of fusion between any spicules. Microscleres are scattered evenly throughout the choanosome. Ectosomal skeleton of the dermal side consists of abundant prostal diactins passing through the distal tangential parts of hypodermal pentactins and dermalia, which are mostly stauractins (62% of 126 assessed), pentactins (29%) and hexactins (10%). The atrial ectosome lacks hypoatrial pentactins but has atrialia in the form of hexactins (89% of 126 assessed), pentactins (8%), stauractins, and triactins (1.5% each). Microscleres are present as in the choanosome.

***Spicules*.** Megascleres (Fig. 3; Table 1) are prostal diactins, hypodermal pentactins, choanosomal diactins, dermalia mostly as stauractins, and atrialia mostly as hexactins. Prostal diactins (Fig. 3A) are long bow-shaped spicules, smooth except for patches of subterminal spines; the smooth tips are rounded or parabolic; the spicule centre is not swollen. Hypodermal pentactins (Fig. 3B) are regular and crucial in form with very long proximal rays, averaging 3.4 × tangential ray length, and fine spines evenly scattered over the entire surface. All five rays have subterminal patches of larger spines and smooth round tips. Choanosomal diactins (Fig. 3C) are straight, bent or more commonly sinuous in shape. Most are broken so few intact spicules are measurable for length. They are smooth except for subterminal inflated rough patches; the tip is smooth and abruptly tapered to a point. The spicule centre is moderately swollen. Dermalia (Fig. 3D) are mainly crucial stauractins completely covered with short, rounded knobs or spines; rays are tapered to a round tip. Atrialia (Fig. 3E) are mostly hexactins ca. half of which are pinular with one ray longer than the others. Like dermalia, these are entirely covered with short, rounded knobs or spines but longer than those of the dermalia; ray tips are rounded.

**Table 1.** Spicule dimensions (µm) of *Bathydoruspoculum* sp. nov., from holotype NIWA 126338.

Parameter	mean	s.d.	range	no.
Dermal prostal diactin				
length (mm)	26.8	11.7	11.0–63.4	48
width	65.6	14.1	16.1–92.6	63
Hypodermal pentactin				
tangential ray length	476	110	218–995	60
tangential ray width	15.3	3.0	8.4–23.2	62
proximal ray length (mm)	1.6	0.6	0.7–4.2	62
proximal ray width	16.5	3.6	9.1–25.8	60
Choanosomal diactin				
length (mm)	16.8	11.1	1.4–31.5	8
width	12.8	7.3	7.1–38.4	47
Dermalia, stauractin				
ray length	98.5	18.0	66.7–139.0	29
ray width	5.0	0.8	3.4–7.7	32
Atrialia, pinular hexactin				50
pinular ray length	150.0	17.4	107.7–181.1	21
pinular ray width	3.8	0.8	2.4–5.2	21
tangential ray length	92.8	10.6	73.1–110.5	21
tangential ray width	3.6	0.6	2.7–4.6	21
proximal ray length	79.4	13.2	62.6–108.0	16
proximal ray width	3.7	0.6	2.7–5.0	20
Atrialia, non-pinular hexactin				
ray length	89.2	9.5	73.6–106.2	21
ray width	3.4	0.7	2.3–4.6	21
Oxy- and hemioxyhexaster				
diameter	109.0	21.8	66.2–164.3	30
primary ray length	4.6	0.9	2.9–7.3	30
secondary ray length	50.4	11.1	26.9–74.5	30
Oxyhexactin				
diameter	119.5	22.2	81.6–157.0	8
ray width	1.5	0.3	1.2–1.9	8

Microscleres (Fig. 3; Table 1) are all oxyhexasters and their variants with hemioxyhexasters being the most common. Oxyhexasters (Fig. 3F, G) have short smooth primary rays and long straight secondary rays; the secondary rays are entirely ornamented with reclined spines that increase in size from the ray tip to its proximal end. Secondary rays on each primary ray vary from 2–5. Hemioxyhexasters (Fig. 3H) are similar to oxyhexasters but at least one of the six primary rays bear only a single secondary ray. Other rare variants include oxyhexactins, oxypentasters, and oxystaurasters (Fig. 3I).

###### Etymology.

Named for the beaker-shaped morphology of this species (*poculum*, beaker; Latin).

###### Remarks.

This New Zealand specimen, NIWA 126338, is entirely consistent with the diagnosis of *Bathydorus* and is assigned there. Each of the known species of the genus differ from this specimen in the following characters: *Bathydorusechinus* Koltun, 1967 has prostal pentactins in addition to diactins, and dermalia as mainly pentactins; *B.fimbriatus* Schulze, 1886 has prostalia including pentactins as marginalia only, and no pinular atrialia; *B.laevislaevis* Schulze, 1886 has no prostalia lateralia and no pinular atrialia; *B.laevispseudospinosus* Tabachnick & Menshenina, 2013 has some large choanosomal or prostal hexactins and smaller oxyhexasters to only 100 µm diameter; *B.laninger* Kahn, Geller, Reiswig & Smith Jr., 2013 has a flat body form and no prostalia on the atrial (upper) surface; *B.servatus* Topsent, 1927 has no prostal diactins, and dermalia as stauractins and diactins; *B.spinosissimus* Lendenfeld, 1915 has choanosomal hexactins, and oxyhexasters with longer primary rays (4–12 µm); in the original description of *B.spinosus* Schulze, 1886, there is no mention of hypodermal pentactins; although Tabachnick and Menshenina (2013) include these, they fail to certify that they are present in the holotype; this species also has wavy secondary rays on the oxyhexasters; *B.uncifer* Schulze, 1899 has smooth dermal and atrial surfaces, and dermalia as mainly pentactins and stauractins. These differences are sufficient to conclude that the new form is a new species, here designated as *Bathydoruspoculum* sp. nov.

##### Nubes

Taxon classificationAnimaliaLyssacinosidaRossellidae

Reiswig, Dohrmann & Kelly gen. nov.

84070042-4F89-55DF-9D7C-A2A896F7A417

http://zoobank.org/032AA823-2695-4E82-888D-0051A86BC438

###### Diagnosis.

Rossellinae with basiphytous, saccular, thick-walled body, unstalked or with a short stalk. Hypodermalia are large, raised, paratropal or orthotropal pentactins with strongly curved or straight tangential rays, smooth except for rough tips, forming a cloud or veil around the thick-walled body. Prostal diactins are marginalia only. Choanosomal spicules are diactins and sometimes large hexactins with curved rays, smooth except for rough tips. Dermalia are mainly stauractins and pentactins. Atrialia are mainly hexactins and sometimes pentactins. Microscleres are oxyhexasters, hemioxyhexasters, and anisodiscohexasters.

###### Etymology.

Named for the cloud of large hypodermal pentactins that veils the body of these sponges (*nubes*, cloud; Latin).

###### Type species.

*Nubestubulata* sp. nov.

###### Remarks.

This new genus diagnosis differs from those of most other anisodiscohexaster-bearing genera or subgenera in the following ways: from *Anoxycalyx* Kirkpatrick, 1907 in not having anchorate hypodermalia, and having pleural hypodermalia raised, having marginalia; in not including pappocomes and discohexasters other than anisodiscohexasters (strobiloidal discohexasters) as microscleres. It differs from that of Crateromorpha (Crateromorpha) Gray in Carter, 1872 in body form, having marginal diactins, and having main atrialia as hexactins. It differs from that of *Rossella* Carter, 1872 in having most atrialia as hexactins instead of stauractins, and no calycocomes. However, it does not differ from the present diagnosis of *Vazella* Gray, 1870 (Tabachnick 2002) in any way, but below we offer a modified diagnosis of that genus to separate the two groups.

##### Nubes tubulata

Taxon classificationAnimaliaLyssacinosidaRossellidae

Reiswig, Dohrmann & Kelly sp. nov.

D0CFA768-826F-5F16-B840-4AB1356975B9

http://zoobank.org/352141EE-D1CC-4A5A-94F5-F52B731D5C73

**Figure 4.**
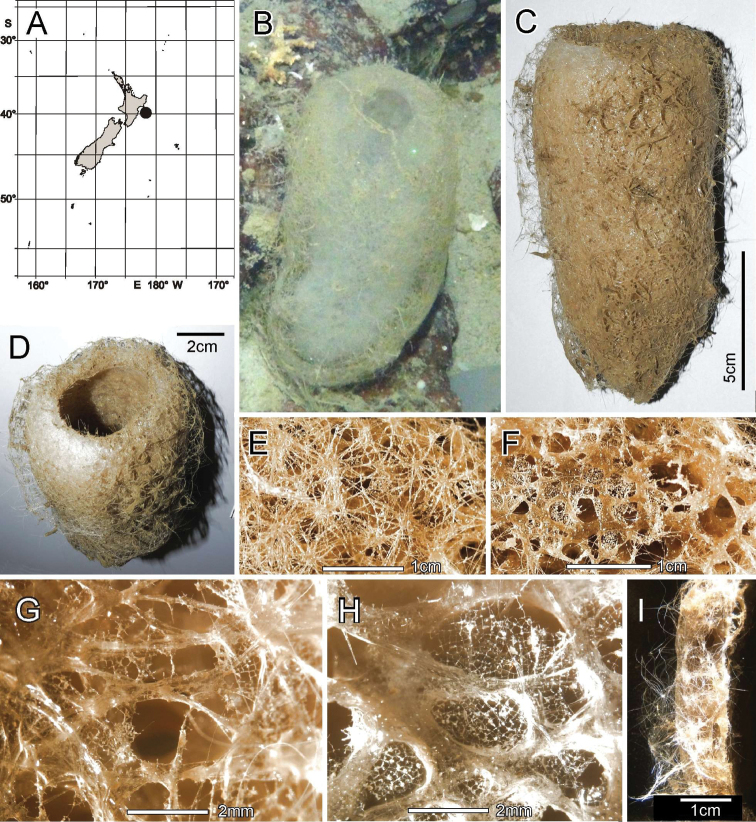
*Nubestubulata* gen. nov., sp. nov., holotype NIWA 126159, distribution, skeleton and morphology **A** Distribution in New Zealand waters **B** holotype in situ **C** holotype, deck image **D** holotype, deck image showing moderate-sized osculum and veil of hypodermal pentactins (deck images by PJS) **E** dermal surface with dense veil of prostal hypodermal pentactins **F** atrial surface without a hypodermal veil **G** closer view of dermal surface with disrupted lattice **H** closer view of atrial surface with intact lattice over exhalant apertures **I** section of body wall, dermal surface on left side. Image **B** captured by ROV Team GEOMAR, ROV Kiel 6000 onboard RV Sonne (voyage SO254), courtesy of Project PoribacNewZ, GEOMAR, and ICBM.

**Figure 5.**
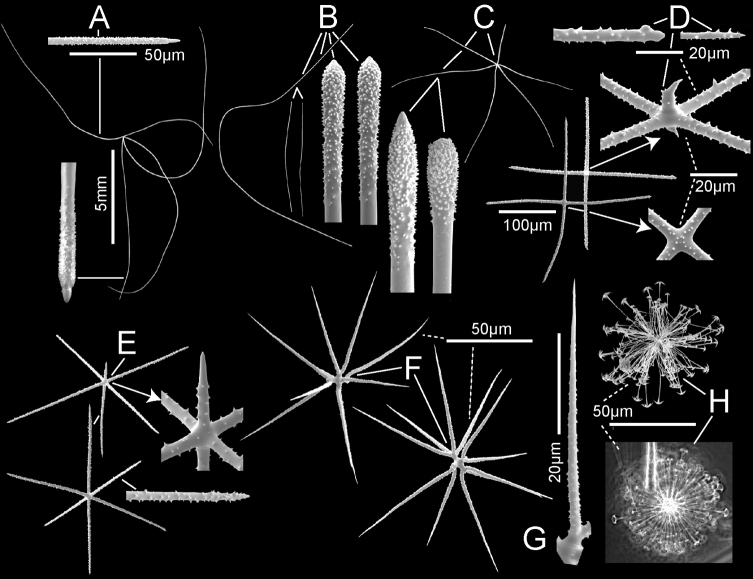
*Nubestubulata* gen. nov., sp. nov., holotype NIWA 126159, spicules **A** whole prostal hypodermal pentactin and enlarged ray ends **B** whole curved and straight choanosomal diactins with two enlarged ends; scales of whole spicules and parts in **B** and **C** as in **A**; **C** whole choanosomal hexactin with two enlarged ray ends **D** two dermalia, a subhexactin, and a stauractin, with enlarged ray ends and centres **E** two atrialia, a pinular subhexactin, and a regular hexactin with enlarged centrum of the pinular subhexactin and a ray end; scales of whole spicules and parts as in **D**; **F** two oxyhexasters **G** enlarged terminal ray of an oxyhexaster **H** anisodiscohexasters, from SEM preparation (above) and LM preparation (below).

Figs 4 , 5 ; Table 2

###### Material examined.

***Holotype***NIWA 126159, RV Sonne Stn SO254/36ROV10_BIOBOX7, Seamount No. 986, off Hawkes Bay shelf, 39.990°S, 178.214°E, 782.8 m, 09 Feb 2017. ***Paratype***NIWA 126160, RV Sonne Stn SO254/36ROV10_BIOBOX10, Seamount No. 986, off Hawkes Bay shelf, 39.989°S, 178.214°E, 767 m, 09 Feb 2017.

###### Distribution.

Known only from the type locality, Seamount 986 off Hawkes Bay shelf, east of North Island, New Zealand (Fig. 4A).

###### Habitat.

Attached to hard substratum; depth 767–783 m (Fig. 4B).

###### Description.

Morphology of the holotype and paratype a thick-walled, tubular sponge, attached to hard substratum by a narrow base (Fig. 4B). A round osculum of moderate size is terminal and opens into a deep atrial cavity. The margin is sharp and there are indications of sparse diactin marginalia in deck images, but we have been unable to verify them in the material at hand. The dermal surface has a dense covering of raised, prostal, hypodermal pentactins (Fig. 4C, D, I) projecting up to 1 cm from the surface proper. There is indication in some of the deck images of long diactins projecting sparsely up to 6 cm from the dermal surface, especially basally, but these may be choanosomal diactins pulled out during collection; we have not found such large diactins in the material we had for examination. Dimensions of the holotype are ~ 13.3 cm high, 7.0 cm wide, and 10.8 (9.2–13.3) (n = 9) mm in body wall thickness; the osculum is 2.2 cm in diameter in situ. The paratype is 19.5 cm high, 13.4 cm wide, and body wall is 7.4 (5.5–9.3) (n = 11) mm in thickness. The osculum is 4.2 cm in diameter in situ. Texture is soft, compressible, and resilient, neither hard nor fragile. Surface of the dermal side is covered by a thick layer of projecting hypodermal pentactins (Fig. 4E). The dermal lattice is torn apart, and dermalia reside in preserved specimens as attached flakes on the hypodermalia (Fig. 4G). The atrial layer retains the atrial lattice covering smaller apertures (Fig. 4F, H); no large megascleres project into the atrium. Colour in life is transparent white, preserved in ethanol is medium brown (Fig. 4C).

***Skeleton*.** Choanosomal skeleton consists of a loose, vacuolar network of thin choanosomal diactins, large choanosomal hexactins, and the thicker proximal rays of the hypodermal pentactins. There is no evidence of fusion between any spicules. Microscleres are scattered evenly throughout the choanosome. Ectosomal skeleton of the dermal side consists of abundant prostal pentactins supporting a delicate lattice of hexactine, pentactine, and stauractine dermalia. The atrial ectosome lacks hypoatrial pentactins but has bands of diactins that support the atrial lattice of hexactins, providing greater support than available to the dermal lattice. Microscleres are present as in the choanosome.

***Spicules*.** Megascleres (Fig. 5; Table 2) are prostal hypodermal pentactins, choanosomal diactins, choanosomal hexactins, dermalia, and atrialia. Prostal hypodermal pentactins (Fig. 5A) are mostly large, raised paratropal forms (90% of 68 scored) with long, very curved tangential rays, but some regular, crucial forms occur (10%) in smaller forms especially near the margin. Tangential rays are 1.7 × the shorter, straighter proximal rays. The spicules are smooth except for the rough sharp tips. Choanosomal diactins (Fig. 5B) are straight or strongly curved, usually with undetectable central swellings; they are smooth except for the rough, slightly inflated tips. Choanosomal hexactins (Fig. 5C) are large forms with strongly curved or nearly straight, nearly equal length rays, which are otherwise similar to those of the hypodermalia. Dermalia (Fig. 5D) are entirely spined and consist of stauractins (31% of 387 scored) and similar forms with reduced fifth ray (subpentactins) or both fifth and sixth rays in one axis (subhexactins) (64%) with a few (1–2%) as tauactins, diactins and paratetractins. It was not possible to differentiate the subpentactins and subhexactins either wet in dishes or mounted spicule microscope slides. Tips are either rounded or more often sharp. Atrialia (Fig. 5E) are entirely spined and mostly subhexactins (71% of 125 scored) with one ray reduced or hexactins (26%) with all rays of nearly equal length; a few (1–2%) are stauractins and tauactins. Ray tips are sharp-pointed.

**Table 2.** Spicule dimensions (µm) of *Nubestubulata* gen. nov., sp. nov. from holotype 126159.

Parameter	mean	s.d.	range	no.
Prostal hypodermal pentactin, lateral body				
tangential ray length (mm)	14.4	1.7	10.5–17.9	36
tangential ray width	42.5	3.3	36.8–50.4	35
proximal ray length (mm)	8.4	1.3	5.3–10.7	26
proximal ray width	46.6	5.0	36.8–59.4	28
Prostal hypodermal pentactin, margin				
tangential ray length (mm)	2.0	1.4	0.6–7.4	32
tangential ray width	20.2	7.0	6.5–39.6	31
proximal ray length (mm)	2.8	1.7	0.8–6.0	25
proximal ray width	21.9	7.1	7.3–43.0	30
Choanosomal diactin				
length (mm)	9.1	5.1	1.6–21.3	35
width	16.4	9.1	4.2–47.0	35
Choanosomal hexactin				
ray length (mm)	5.9	1.9	2.5–10.9	46
ray width	39.0	8.9	21.0–60.7	45
Dermalia stauractin				
ray length	132	17	91–174	36
ray width	5.7	0.8	4.5–7.3	20
Dermalia subpentactin/hexactin				
ray length	142	17	107–180	36
ray width	5.4	0.8	4.2–7.1	20
Atrialia subhexactin short pinular				
ray length	21	5	13–40	26
tangential ray length	176	25	130–230	28
proximal ray length	130	22	93–184	26
tangential ray width	5.8	0.9	4.1–7.8	26
Atrialia, non-pinular hexactin				
ray length	171	17	139–220	27
ray width	5.8	1.1	3.8–8.0	26
Oxy- and hemioxyhexaster				
diameter	130.5	14.1	90.2–165.7	32
primary ray length	5.3	1.1	3.7–8.9	32
secondary ray length	60.2	6.2	47.3–77.1	32
Anisodiscohexaster				
diameter	70.9	7.3	47.5–81.7	35
primary ray length	5.5	0.9	4.0–7.5	35
longest secondary ray length	30.3	3.9	19.4–36.0	35

Microscleres (Fig. 5; Table 2) are oxyhexasters and their variants, with hemioxyhexasters being the most common, and anisodiscohexasters. Oxyhexasters and hemioxyhexasters (Fig. 5F, G) have very short smooth primary rays and long straight secondary rays entirely ornamented with small, reclined spines. Secondary rays on each primary ray vary from 1–4. Anisodiscohexasters (Fig. 5H) are spherical with stellate discs with 4–6 marginal claws on the ends of terminal rays. Primary rays are smooth and end in strobiloid discs with a short central projecting knob. Each primary strobilum supports 30–40 terminal rays with undulating, probably helically coiled, finely rough shafts of unequal lengths. Terminal discs vary in diameter with shaft length, the longer shafts carrying the larger discs, e.g., a series 1.7, 2.5, 3.1, 3.4, 3.6, 5.4, 6.9 µm diameter for shafts 15.0, 20.5, 23.5, 27.3, 32.0. 33.4, 37.1 µm in length. These spicules look very different in LM (lower image) and SEM (upper image) due to collapse of the rays during drying for SEM and support of them by balsam in LM.

###### Etymology.

Named for the tubular morphology of the sponge (*tubulata*, tubular; Latin).

###### Remarks.

The characters of these two New Zealand specimens are inconsistent with the present diagnoses of all Rossellinae genera except *Vitrollula* Ijima, 1898. They differ, however, from those of *V.fertilis* Ijima, 1898, the only species in the genus, in characters not used as diagnostic. These are that *V.fertilis* has a smooth surface without raised hypodermalia, but the two new specimens have a bristly surface with raised hypodermalia, and that the discohexasters of *V.fertilis* are isodiscohexasters while those of the new species are anisodiscohexasters. In view of these differences, we opt not to include the new species in *Vitrollula* nor to change the diagnosis of that genus at this time. We choose to erect a new genus in Rossellinae with characters of this and the following second species described below, and designate this species as *Nubestubulata* gen. nov., sp. nov.

##### Nubes poculiformis

Taxon classificationAnimaliaLyssacinosidaRossellidae

Reiswig, Dohrmann & Kelly sp. nov.

BD8B043A-48B9-551B-AEB1-EEE18E9F7785

http://zoobank.org/2EBDD0FB-6EB9-498A-8749-595C64824C23

**Figure 6.**
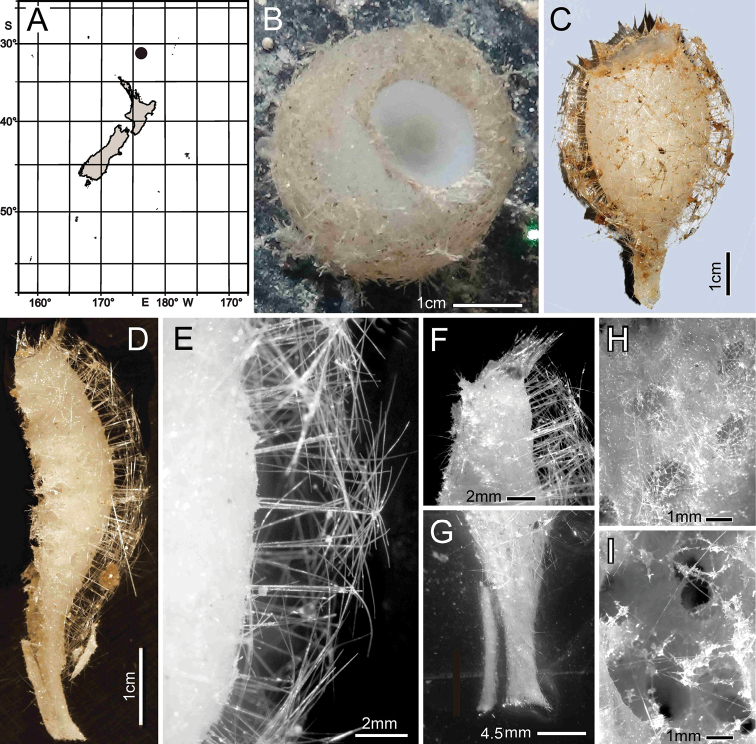
*Nubespoculiformis* gen. nov., sp. nov., holotype NIWA 126016, distribution, skeleton and morphology **A** distribution in New Zealand waters **B** holotype in situ **C** holotype, deck image (by PJS); **D** longitudinal section of holotype, showing hypodermal pentactin veil **E** closer view of hypodermal pentactin veil **F** edge of osculum with tuft of marginalia **G** close view of stalk subdivided for spicule preparation of smaller sample **H** dermal surface with intact lattice of dermalia over inhalant canals **I** atrial surface with disrupted lattice of atrialia. Image **B** captured by ROV Team GEOMAR, ROV Kiel 6000 onboard RV Sonne (voyage SO254), courtesy of Project PoribacNewZ, GEOMAR, and ICBM.

**Figure 7.**
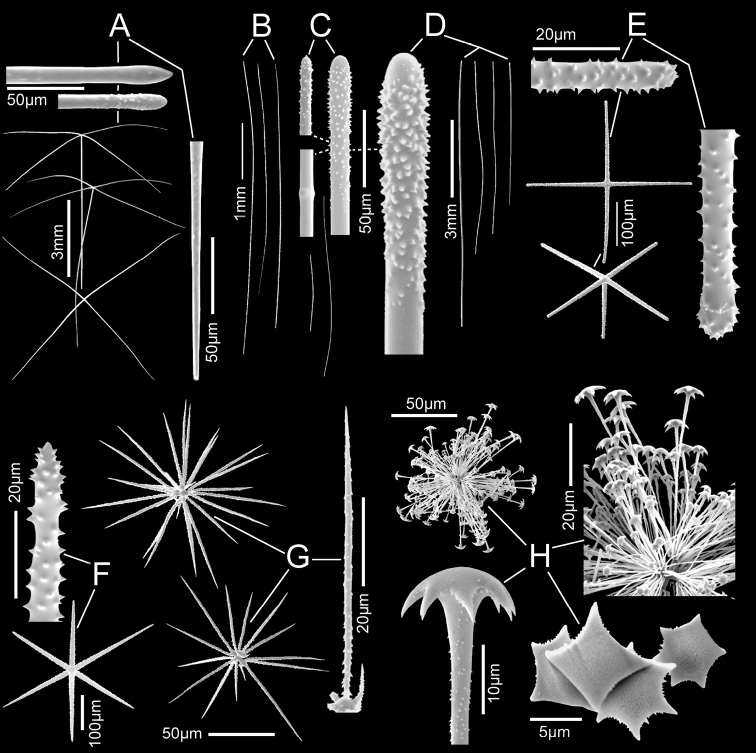
*Nubespoculiformis* gen. nov., sp. nov., holotype NIWA 126016, spicules **A** three prostal hypodermal pentactins, the lower one in plane of tangential rays, with enlarged ray ends **B** whole marginal diactins (ray ends unavailable) **C** two whole choanosomal hexactins with two enlarged ray ends and one centrum. Scale of whole spicules as in **C**; **D** four whole stalk diactins and enlarged end **E** dermalia, stauractin and pentactin with enlarged ray ends **F** atrialium and enlarged ray end **G** two oxyhexasters and enlarged terminal ray **H** whole anisodiscohexaster; an enlarged section showing disc diameter increasing in longer terminal rays; an enlarged side view of a terminal ray and end views of terminal ray discs.

Figs 6 , 7 ; Table 3

###### Material examined.

***Holotype***NIWA 126016, RV Sonne Stn SO254/08ROV02_BIOBOX10, Seamount No. 114 in International Waters to the east of Three Kings Ridge and Norfolk Island, 31.301°S, 175.197°E, 1285 m, 31 Jan 2017.

###### Distribution.

Known only from the type locality, Seamount No. 114, in International Waters to the east of Three Kings Ridge and Norfolk Island (Fig. 6A).

###### Habitat.

Attached to hard substratum; depth 1285 m (Fig. 6B).

###### Description.

Morphology of the holotype body is a thick-walled tubular sponge, attached to hard substratum, by a moderately long, narrow stalk (Fig. 6B, C). A moderately sized, round osculum is terminal and opens into a deep atrial cavity. The margin is blunt, bordered by a band of diactine marginalia (Fig. 6D, F). The dermal surface has a dense covering of raised, prostal, hypodermal pentactins (Fig. 6D, E), projecting up to 7 mm from the surface proper. Some of the deck images indicate long diactins projecting sparsely, up to 14 mm, from the dermal surface, but these may be foreign in origin; we have not found such large diactins in the material available for examination. Dimensions of the holotype are ~ 6 cm in total length, including the stalk of 1.8 cm length (Fig. 6G), and 3.5 cm in width; the maximum body wall thickness is 13.9 mm. The osculum is 12.3 by 16.9 mm diameter in situ. Texture is soft, compressible, and resilient, neither hard nor fragile. Surface of the dermal side below the layer of projecting hypodermal pentactins is supported by an intact tight lattice of dermalia (Fig. 6H). The atrial surface (Fig. 6I), in contrast, is torn apart by removal from supporting fluids and the atrial lattice remains only as dismembered patches attached to underlying diactins. Colour in life is pale brown as is the specimen preserved in ethanol.

***Skeleton*.** Choanosomal skeleton consists of a loose, vacuolar network of thin choanosomal diactins, large choanosomal hexactins and the thicker proximal rays of the hypodermal pentactins. There is no evidence of fusion between any spicules. Microscleres are scattered evenly throughout the choanosome. Ectosomal skeleton of the dermal side consists of abundant prostal pentactins providing good support for the sturdy lattice of stauractine (60.0% of 315 assessed), pentactine (38.4%), and rare hexactine (1.64%) dermalia. The atrial ectosome lacks hypoatrial pentactins but has bands of diactins that provide poor support for the atrial lattice of mainly hexactins (86.4% of 118 assessed), pentactins (7.5%), and stauractins (5.1%). Microscleres are present as in the choanosome.

***Spicules*.** Megascleres (Fig. 7; Table 3) are prostal hypodermal pentactins, marginal diactins, choanosomal diactins of the body, choanosomal diactins of the stalk, dermalia and atrialia. Prostal hypodermal pentactins (Fig. 7A) are large, raised orthotropal forms with long straight tangential rays. Tangential rays are ca. one half the length of the longer straight proximal rays. The spicules are smooth except for the rough sharp or round tips. Marginalia (Fig. 7B) are long, slightly curved diactins; no intact tips were found in SEM surveys but an exhaustive survey with LM indicates tips taper to nearly invisible thinness and are quite distinct from the thick roughened tips of choanosomal diactins. Choanosomal diactins (Fig. 7C) are straight or slightly curved with undetectable central swellings; they are smooth except for the rough, slightly inflated tips. Stalk diactins (Fig. 7D) are longer and thicker than the choanosomal diactins, but otherwise similar. Dermalia (Fig. 7E) are mainly entirely rough stauractins and pentactins with rounded ray tips. Atrialia (Fig. 7F) are entirely rough hexactins with equal length rays and more acute ray tips.

**Table 3.** Spicule dimensions (µm) of *Nubespoculiformis* gen. nov., sp. nov. from holotype 126016.

Parameter	mean	s.d.	range	no.
Prostal hypodermal pentactin				
tangential ray length (mm)	3.9	0.8	1.9–5.2	73
tangential ray width	51.1	5.4	36.4–59.3	60
proximal ray length (mm)	6.5	1.1	3.3–8.0	69
proximal ray width	51.0	4.5	39.7–58.7	59
Marginal diactin				
length (mm)	4.5	0.7	2.8–6.0	58
width (mm)	18.0	3.3	12.3–27.5	64
Choanosomal diactin				
length (mm)	2.5	1.4	0.6–4.9	52
width (mm)	11.8	2.3	7.5–19.2	52
Stalk diactin				
length (mm)	7.4	2.1	2.5–11.5	25
width (mm)	14.7	4.9	8.1–29.6	25
Dermalia stauractin ray				
length	200	24	130–243	51
width	11.5	1.7	7.3–14.6	51
Dermalia pentactin tangential ray				
length	185	19	146–223	62
width	11.3	1.5	6.3–15.5	63
Dermalia pentactin proximal ray				
length	155	20	119–190	21
width	11.0	1.6	7.8–14.5	23
Atrialia hexactin				
ray length	227	25	176–283	50
ray width	13.9	2.2	9.2–20.0	50
Oxyhexaster				
diameter	137	11	103–165	51
primary ray length	5.7	1.1	3.5–7.8	51
secondary ray length	62.6	4.9	46.7–72.9	51
Anisodiscohexaster				
diameter	148	34	87–201	54
primary ray length	9.0	1.4	6.0–12.6	54
longest secondary ray length	66.4	16.2	32.9–89.5	54

Microscleres (Fig. 7; Table 3) are oxyhexasters, hemioxyhexasters, and anisodiscohexasters. Oxyhexasters (Fig. 7G) and hemioxyhexasters have very short, sparsely spined or smooth, thick primary rays, ending in swollen hemispheres; 1–7, usually 3–4, rough, straight, terminal rays tapering to pointed tips emanate from the margins and occasionally from the centre of the hemisphere. Short to very short spur-like terminal rays are common. Anisodiscohexasters (Fig. 7H) have smooth primary rays ending in ovoid strobila. Each strobilum supports ca. 20–30 rough, curved terminal rays that end in discs with 4–7 marginal discs. The tuft of terminal rays from each primary ray varies in length of rays, and with ray length the diameter of terminal discs, in a pattern that is not yet clear, but the whole spicule resembles a radially symmetrical starburst.

###### Etymology.

Named for the goblet shape of the sponge (*poculiformis*, goblet-shaped; Latin).

###### Remarks.

This species differs from *Nubestubulata* sp. nov. in having a short stalk and orthotropal hypodermal pentactins, but is otherwise similar enough to include it in the genus *Nubes* as its second species, *Nubespoculiformis* sp. nov.

##### Vazella

Taxon classificationAnimaliaLyssacinosidaRossellidae

Gray, 1870

393B206E-CD43-5721-A5CF-E8111EB41539

###### Diagnosis.

Body is saccular, basiphytous. Choanosomal skeleton is composed of diactins. Hypodermal pentactins are raised, thorned paratropal pentactins. Prostalia basalia and marginalia are monaxons (diactins). Dermalia are stauractins and pentactins. Atrialia are mainly hexactins. Discoid microscleres are microisodiscohexasters and microanisodiscohexasters; oxyoid microscleres are combinations of hexactins, hexasters, and hemihexasters (modified from Tabachnick 2002).

###### Remarks.

This modified diagnosis allows separation of the present genus, *Nubes* gen. nov., from *Vazella* on the basis of lack of thorned hypodermalia and presence of discoid microscleres that are not anisodiscohexasters in the former. Furthermore, molecular phylogenetic results do not support a close relationship of the two genera (MD, unpubl. results).

##### Scyphidium

Taxon classificationAnimaliaLyssacinosidaRossellidae

Schulze, 1900

47AD0383-79F7-5B26-B545-236AD6F2F5D1

###### Diagnosis.

Body is saccular, basiphytous, sometimes rhizophytous. Choanosomal skeleton is composed of diactins. Hypodermal spicules, if present, are pentactins. Prostalia, if present, are hypodermal pentactins and/or diactins. Dermalia are stauractins and/or pentactins in various combinations. Atrialia are mainly hexactins. Microscleres are discohexasters and oxyhexasters often with hemioxyhexasters and oxyhexactins; with two or three types of discohexasters, none as calycocomes. Among the larger is a spherical form with a restricted number of secondary rays (emended from Tabachnick 2002).

###### Remarks.

The genus diagnosis is emended of necessity, to accept *S.australiense* Tabachnick, Janussen & Menschenina, 2008 and *S.variospinosum* sp. nov., described below.

###### Type species.

*Scyphidiumseptentrionale* Schulze, 1900.

##### Scyphidium australiense

Taxon classificationAnimaliaLyssacinosidaRossellidae

Tabachnick, Janussen & Menschenina, 2008

44B4A7D7-A373-5F1C-8A3E-1ABDD7800305

**Figure 8.**
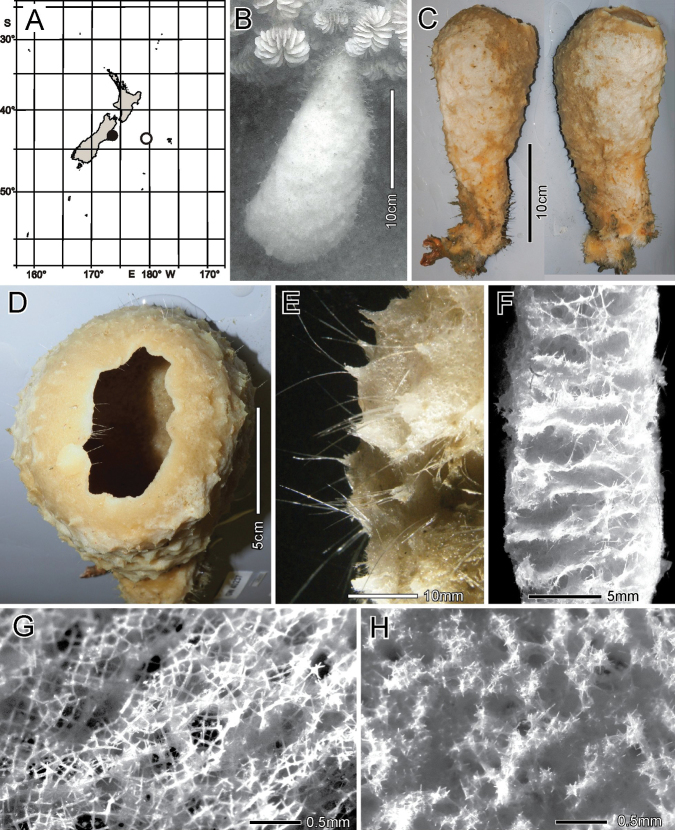
*Scyphidiumaustraliense* Tabachnick, Janussen & Menschenina, 2008, NIWA 126237, distribution, skeleton and morphology **A** distribution in New Zealand waters, holotype as open circle, new specimen as filled circle **B** new specimen in situ (scale bar is approximate) **C** deck image (two sides, image by PJS) **D** osculum, deck image (by PJS) **E** preserved conulose outer surface of the lower body with prostal diactins **F** preserved wall section of the mid-body without conules **G** preserved dermal surface with intact pentactin lattice **H** preserved atrial surface with hexactins displaced from the atrial lattice. Image **B** captured by ROV Team GEOMAR, ROV Kiel 6000 onboard RV Sonne (voyage SO254), courtesy of Project PoribacNewZ, GEOMAR, and ICBM.

**Figure 9.**
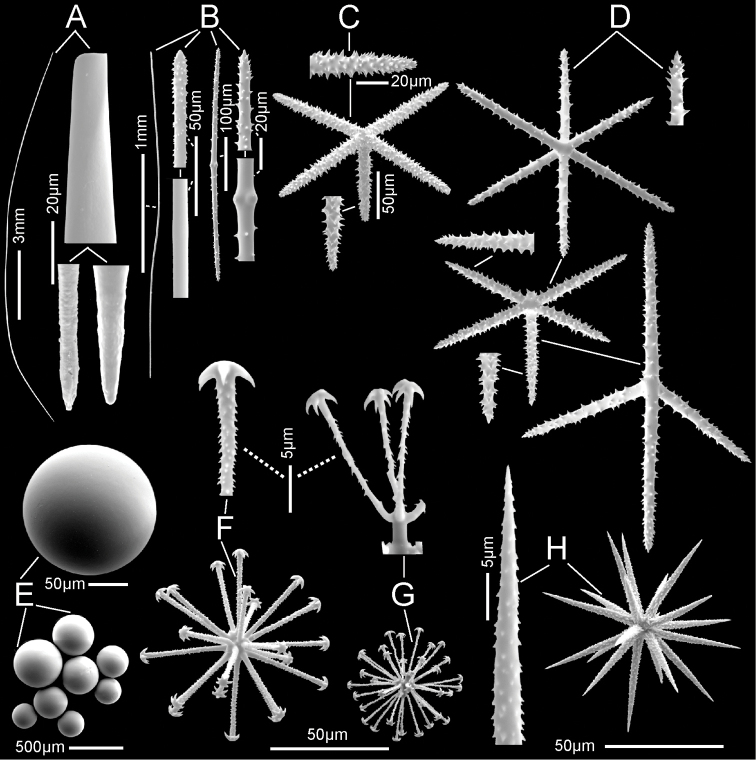
*Scyphidiumaustraliense* Tabachnick, Janussen & Menschenina, 2008, NIWA 126237, spicules **A** prostal diactin, whole and enlarged ends, one broken distal end and two intact proximal ends **B** choanosomal diactins, whole long and short versions at different scales plus enlarged tips and central swellings **C** dermalium: pentactin, whole and enlarged tips **D** atrialia, hexactin, whole and enlarged tip, pentactin, whole with enlarged tips, and paratetractin, whole; scales are the same as those for dermalium **E** spheres as small group of whole ones and one enlarged **F**discohexaster 1, whole and enlarged terminal ray end **G**discohexaster 2, whole and enlarged part of one ray tuft **H** oxyhexaster, whole and enlarged terminal ray end. Scales are the same for all whole microscleres and their enlarged parts.

Figs 8 , 9 ; Table 4

###### Note.

From the ending of its name, *Scyphidium* is a neuter noun, and thus *S.australiensis* (as originally named by Tabachnick et al. 2008) should be *S.australiense*. This is borne out by the names of conspecifics that are also adjectives (e.g., *S.chilense*, *S.septentrionale*, *S.tuberculatum*) (J. Rosser, pers. comm.). We hereby make that change and use the corrected name throughout this work.

###### Type and locality (not examined).

Holotype – NIWA 155561, RV Sonne Stn SO17/80 (NZOI Stn Z3951B), Chatham Rise, 43.553°S, 179.457°E, 409 m, 10 Apr 1981 [Originally cited in Tabachnick et al. (2008) as WAM (p14), RV Soela Stn SO 17–80, 43°33.10'–33.05'S, 179°27.25'–27.08'E, depth unknown].

###### Material examined.

NIWA 126237, RV Sonne, Stn SO254/77ROV14_BIOBOX02, Pegasus Canyon slope, off Christchurch shelf, 43.2927361°S, 173.6066742°E, 853 m, 20 Feb 2017.

###### Distribution.

Chatham Rise and Pegasus Canyon slope, off Christchurch shelf Christchurch shelf, New Zealand (Fig. 8A).

###### Habitat.

Attached to hard substratum; depth 409–853 m.

###### Description.

Body form is a heavy-looking, thick-walled, club-shaped, pendant sponge with a narrow basal attachment, widening gradually to a hemispherical rounded terminal end (Fig. 8B, C) where a large osculum is centrally located. The osculum opens into a deep atrial cavity (Fig. 8D). The margin is sharp-edged with indication of sparse marginalia that do not differ from prostal diactins of the lower body. The external surface of the upper body is fairly smooth, without prostalia, but the lower half is conspicuously conulose with long prostal diactins projecting in small groups from conules (Fig. 8E). We did not have access to the basal attachment so we cannot comment on the basidictyonalia. Dimensions of the specimen are 27.6 cm in height, 11.7 cm in maximum width, 5.7–10.9 cm in diameters of the osculum, 10.0 mm in maximum wall thickness, 8.3 mm in length of projecting part of prostal diactins. Texture is firm but compressible and resilient, neither soft nor fragile. Surface of the dermal side is covered by an intact lattice of dermalia (Fig. 8G) consisting mostly of pentactins (98% of 302 assayed), and a few stauractins and diactins (1% each). The upper body surface is fairly smooth, but the lower body is covered with conspicuous conules up to 3.2 mm high, from which prostal diactins project in small groups of one to four. One large pentactin was found but it was broken and assumed to be foreign. The atrial surface is covered by a felt-like layer of disarranged atrialia (Fig. 8H) composed of hexactins (57% of 168 assayed), pentactins (20%), paratetractins (8%), diactins (6%), stauractins (5%), and triactins (3%). Colour in life is very pale brown, preserved in ethanol is medium brown.

***Skeleton*.** Choanosomal skeleton consists of a tight series of macroscopic partitions of inhalant and exhalant channels running perpendicular to the body surfaces (Fig. 8F). They consist of networks of choanosomal diactins and microscleres and in the lower body the proximal ends of the prostal diactins. A few small patches of fused choanosomal diactins occur but these are too rare to provide significant support to the body. Ectosomal skeleton of the dermal side consists of the robust lattice of pentactine dermalia and in the lower body the projecting prostal diactins. The atrial ectosomal skeleton consists of the felt-like lattice of atrialia and the supporting layer of hypoatrial diactins.

**Spicules.** Megascleres (Fig. 9; Table 4) are prostal diactins, choanosomal diactins, dermalia, and atrialia. Prostal diactins (Fig. 9A) are large, curved, and smooth spicules with rounded proximal tips either smooth or bearing very low suggestions of obsolete spines. They have neither an axial cross nor central swellings. Distal tips are invariably broken off. Choanosomal diactins (Fig. 9B) come in three distinct forms. The larger ones over 2 mm long are straight or slightly curved or sinuous and are smooth except for the patches of spines at the rounded or abruptly pointed tips. Those between 1 and 2 mm long have sharp tips and longer spines on the tip patches. The shortest, less than 1 mm long, are entirely spined with sharp tips and often with a central tyle or four knobs. Dermalia (Fig. 9C) are thick stubby pentactins, entirely profusely spined without a knob of a sixth ray. Atralia (Fig. 9D) are highly diverse; the most common hexactins have thinner and less densely spined rays than the dermalia. Pentactin atrialia are very similar to the dermal pentactins but have a knob in place of the sixth ray. Paratropal atrialia have rays similar to the hexactine atrialia. Spheres (Fig. 9E) are common and here considered megascleres.

**Table 4.** Spicule dimensions (µm) of *Scyphidiumaustraliense* Tabachnick, Janussen & Menschenina, 2008 from holotype NIWA 126237.

**Parameter**	**mean**	**s.d.**	**range**	**no.**
Prostal diactin				
length (mm)	10.9	3.9	5.7–18.3	31
width	83.9	27.7	37.8–172.3	46
Choanosomal diactin				
length (mm)	2.0	1.3	0.4–4.4	38
width	13.1	3.6	6.1–21.7	50
Dermalia pentactin				
tangential ray length	145	17	106–186	31
ray width	15.3	1.8	11.0–18.4	31
proximal ray length	119	19	57–165	31
ray width	14.4	1.8	12.0–18.2	31
Atrialia hexactin				
ray length	206	80	88–359	40
ray width	14.3	3.4	7.7–24.5	40
Sphere				
diameter	189	77	90–388	54
Discohexaster 1				
diameter	69.8	10.2	50.0–91.2	32
primary ray length	4.8	0.7	3.4–6.8	32
secondary ray length	30.3	5.4	20.6–42.8	32
Discohexaster 2				
diameter	50.2	10.0	33.4–79.4	68
primary ray length	4.8	0.9	2.7–7.0	68
secondary ray length	20.3	4.9	11.7–34.6	68
Oxyhexaster				
diameter	86.2	10.6	63.5–111.3	59
primary ray length	5.6	1.2	3.2–9.0	59
secondary ray length	37.3	5.5	23.8–49.8	59

Microscleres (Fig. 9; Table 4) are two types of discohexasters and one type of oxyhexaster and its variants, rare hemioxyhexasters and oxyhexactins. Discohexasters 1 (Fig. 9F) are spherical with very short smooth primary rays, each supporting 3.5 (2–5) thick secondary rays ornamented with reclined spines. Terminal discs invariably have six stout marginal teeth. Discohexasters 2 (Fig. 9G) are smaller spherical forms with each smooth primary ray supporting 6.3 (5–8) thinner terminal rays; the terminal discs also invariably have 6 marginal teeth. Oxyhexasters (Fig. 9H) are stout spherical forms with each short smooth primary ray supporting 3.2 (3–5) fully developed secondary rays ornamented with dense reclined spines and ending in sharp tips. Each oxyhexaster also has 2–12 poorly developed secondary rays only a few micrometres in length. Only one hemioxyhexaster and three oxyhexactins, all of a similar size and ray characters as the oxyhexaster, were discovered in microsclere surveys.

###### Remarks.

The characters of this new specimen agree with those in the original description of *S.australiense* by Tabachnick et al. (2008) except for the absence of prostal diactins and sphere megascleres in the latter, and absence of the rare discohexactins in the former. Absence of prostal diactins in the holotype is likely attributable to it being a distal fragment where we also found no prostalia in the new specimen. Spheres appear to be spicules of erratic occurrence in hexactinellids and are unlikely to be of phylogenetic significance. Absence of discohexactins in the new specimen is not considered an important difference. Sizes and shapes of the common microscleres are similar enough in both specimens to conclude that they are from specimens of the same species. It is somewhat surprising that the authors of this species assigned it to the genus *Scyphidium* without altering the generic diagnosis to encompass it; we have done so here.

Prior to the discovery of a second specimen of *S.australiense* here, there was considerable doubt as to the true type locality of the holotype described by Tabachnick et al. (2008). This work focused on hexactinellid sponges “sampled mainly off the Australian West Coast”, and the holotype was named “after the type locality of this species”, i.e., Australia. However, the latitude and longitude for RV Soela Stn SO 17–80 (43°33.10'–33.05'S, 179°27.25'–27.08’E) placed the type locality as on the north central Chatham Rise on the east coast of New Zealand. The Western Australian Museum (WAM) has confirmed that the RV Soela carried out fieldwork off western and northern Australia, and that the material covered in Tabachnick et al. (2008) was sent to the MNHN to be worked on taxonomically. Unfortunately, WAM has no details for “RV Soela Stn SO 17–80" (Jane Fromont, Western Australian Museum, pers. comm.), but interestingly, the specimen reported here, NIWA 126237, is also from Chatham Rise (Pegasus Canyon Slope, off Christchurch Shelf), intensifying the mystery surrounding the type locality of this species. Investigation of pre-2004 electronic records at NIC revealed that the specimen listed from station “RV Soela Stn SO 17–80", given in Tabachnick et al. (2008), was more likely to have been collected on the RV Sonne Cruise SO-17 on the Chatham Rise phosphorite deposits east of New Zealand (Von Rad 1984), because the NZOI Stn Z3951B from that cruise, a large grab with Porifera listed in the Remarks column, has identical coordinates and similar station numbers. We are still unsure as to how the specimen reached Tabachnick’s attention at the MNHN, and indeed, the whereabouts of the holotype, but we know that errors were made in translation of the station data from the specimen labels to this publication, and it is possible that the authors assumed that the RV Sonne representation of “Stn SO17/80" was just another RV Soela Stn, represented as SO 17–80 in the publication. The MNHN was temporarily closed for most of 2020 and the early months of 2021 due to measures of the French government to prevent the spread of COVID-19 (novel coronavirus disease), so the repatriation of this specimen was not able to be completed at time of publication.

##### Scyphidium variospinosum

Taxon classificationAnimaliaLyssacinosidaRossellidae

Reiswig, Dohrmann & Kelly sp. nov.

0984F0D2-7E07-5AFA-A164-DCFAC8FF3BB2

http://zoobank.org/6CB20C98-6A3E-41E3-B8A3-C9410668207D

**Figure 10.**
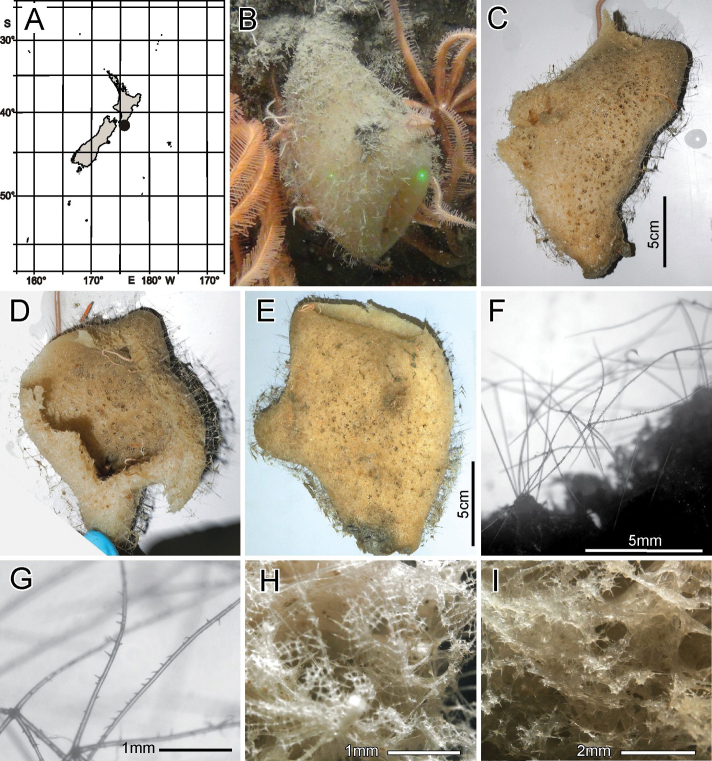
*Scyphidiumvariospinosum* sp. nov.: **A** distribution in New Zealand waters, location of both holotype NIWA 126279 and paratype NIWA 126274 on Wairarapa Slope **B** holotype NIWA 126279 in situ (green laser spots are 6.24 cm apart) **C** holotype, deck image, torn open on the left side. Note the distinct pentactin veil around body **D** holotype, superior end, deck image, where torn wall is obvious, and osculum is partly intact on the upper left side. Scale bar unavailable **E** paratype, NIWA 126274 (deck images by PJS) **F** close view of the prostal pentactins forming the veil of the holotype **G** Closer view of the thorns on the prostal pentactin tangential rays **H** dermal surface of preserved holotype with partly damaged lattice of dermalia **I** atrial surface of the preserved holotype with no lattice evident. Image **B** captured by ROV Team GEOMAR, ROV Kiel 6000 onboard RV Sonne (voyage SO254), courtesy of Project PoribacNewZ, GEOMAR, and ICBM.

**Figure 11.**
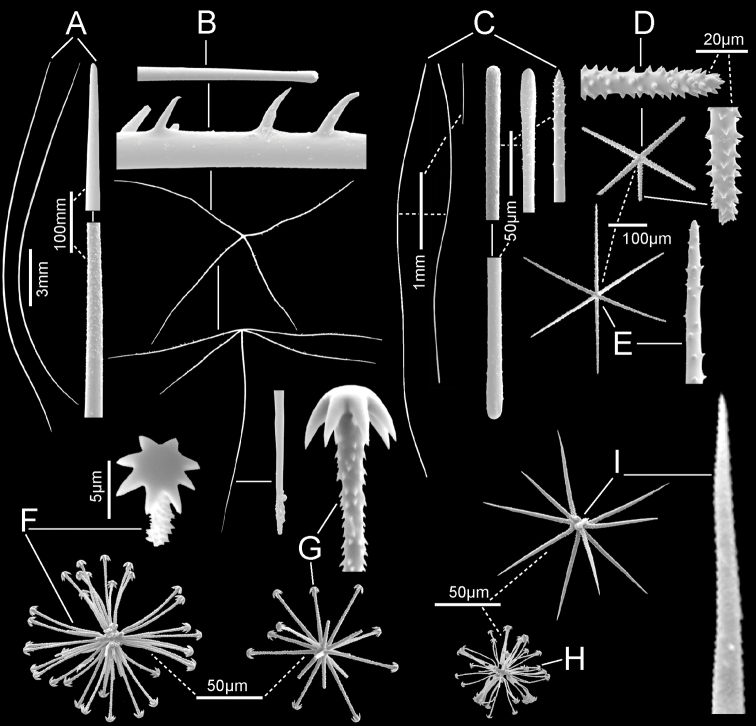
*Scyphidiumvariospinosum* sp. nov., holotype NIWA 126279, spicules **A** prostal diactins, two whole and enlarged end and near-end segment **B** prostal pentactins, two whole spicules, inside and end views and parts including a tangential ray end (top), a thorned part and a proximal end (lower). Scale bars of whole and parts are same as in **A C** choanosomal diactins, three whole long and short versions plus enlarged tips **D** one pentactine dermalium and enlarged tangential and proximal ray tips **E** one hexactine atrialium and enlarged ray end **F**discohexaster 1 and enlarged terminal ray end **G**discohexaster 2 with enlarged terminal ray end **H**discohexaster 3 **I** hemioxyhexaster with enlarged terminal ray end. All microscleres and their enlarged parts are at same scale bars.

Figs 10 , 11 ; Table 5

###### Material examined.

***Holotype***NIWA 126279, RV Sonne Stn SO254/78ROV15_BIOBOX3–5, Wairarapa Slope, 41.619°S, 175.788°E, 1630.5 m, 21 Feb 2017. ***Paratype***NIWA 126274, RV Sonne Stn SO254/78ROV15_BIOBOX1, Wairarapa Slope, 41.619°S, 175.788°E, 1675.1 m, 21 Feb 2017.

###### Distribution.

Known only from two locations on the Wairarapa Slope, New Zealand (Fig. 10A).

###### Habitat.

Attached to hard substratum; depth 1631–1675 m (Fig. 10B).

###### Description.

Morphology of the holotype and paratype is a thick-walled, club-shaped sponge with a narrow basal attachment, widening gradually to a hemispherical rounded terminal end where a large osculum is centrally located (Fig. 10B–E). The osculum opens into a deep atrial cavity. The margin is sharp-edged with indication of sparse marginalia that do not differ from prostal diactins of the general body wall. The external surface of the entire body is clean and conulose, with prostal diactins and hypodermal pentactins emanating from conules in small groups of 1–10. Dimensions of the holotype and paratype are, respectively, length 16.5 and 15.8 cm, width 9.5 and, excluding the lateral bulge, 9.5 cm, diameter of the osculum 6.4 and 6.6 cm, wall thickness 11.1 and 10.0 mm. Texture is soft, compressible, and resilient, but not fragile. Surface of the dermal side is conulose and bears a layer of prostal diactins projecting 12.5 (6.5–19.3) (n = 9) mm above the dermal surface, intermingled with a veil of prostal, hypodermal, thorned, paratropal pentactins (Fig. 10F, G) projecting 8.6 (7.2–10.0) (n = 9) mm above the surface. On the surface below is a lattice of overlapping dermalia of mostly pentactins (94% and 82% of 160 and 101 assessed) (Fig. 10H). The atrial surface bears a poorly preserved atrial lattice of mostly hexactins (94.7% and 94% of 133 and 131 assessed) (Fig. 10I) forming a cover over the exhalant apertures. Colour in life and preserved is very pale brown.

***Skeleton*.** Choanosomal skeleton is composed of choanosomal diactins without detectable macroscopic or microscopic organisation. No evidence of spicule fusion was noted in either specimen. Microscleres are scattered throughout the choanosome. Ectosomal skeleton of the dermal side consists of the prostal diactins and projecting veil of thorned pentactins. The dermal surface is covered by a robust lattice of mostly pentactine dermalia. The atrial ectosome consists of the felt-like disorganised lattice of mostly hexactine atrialia and the supporting layer of hypoatrial diactins.

***Spicules*.** Megascleres (Fig. 11; Table 5) are prostal diactins, prostal hypodermal pentactins, choanosomal diactins, dermalia, and atrialia. Prostal diactins (Fig. 11A) are large, curved, and smooth spicules with rough subterminal patches and round tips. They have neither axial crosses nor central swellings. Prostal hypodermal pentactins (Fig. 11B) are large spicules with proximal rays ~ 1.4 times the length of tangential rays; the tangential rays are mainly paratropal, occasionally orthotropal. Both tangential and proximal rays have smooth rounded tips, but only tangential rays bear large thorns on the middle halves of the rays. Choanosomal diactins (Fig. 11C) occur in two distinct forms. Large ones > 2 mm in length are straight, slightly curved or sinuous, and are smooth except for rough subterminal areas; tips are rounded or abruptly pointed. Small forms < 2 mm in length are entirely spined, have sharp tips and often four central knobs or a single tyle. Dermalia (Fig. 11D) are thick stubby pentactins, entirely and profusely spined without a knob of a sixth ray. Atralia (Fig. 11D) are most commonly hexactins, with rays longer, thinner, and less profusely spined than those of dermalia.

**Table 5.** Spicule dimensions (µm) of *Scyphidiumvariospinosum* sp. nov. from holotype NIWA 126279.

Parameter	mean	s.d.	range	no.
Prostal diactin				
length (mm)	15.8	3.9	8.3–22.9	22
width	76.0	18.5	34.0–104.6	30
Prostal pentactin				
tangential ray length (mm)	6.4	1.4	1.0–9.1	36
tangential ray width	77.6	11.3	38.4–94.5	32
proximal ray length (mm)	9.1	1.5	3.8–10.9	31
proximal ray width	91.8	12.0	54.6–109.4	32
Choanosomal diactin				
length (mm)	2.5	1.4	0.4–5.5	32
width	9.5	2.5	5.6–14.4	40
Dermalia pentactin				
tangential ray length	188	21	140–238	53
tangential ray width	14.6	2.2	11.0–18.6	47
proximal ray length	143	21	79–188	45
proximal ray width	14.0	1.9	8.3–18.2	47
Atrialia hexactin				
ray length	275	40	181–384	47
ray width	10.1	1.7	6.2–12.9	49
Discohexaster 1				
diameter	116	19	67–142	33
primary ray length	8.4	1.1	6.2–10.5	33
secondary ray length	49.7	9.3	27.2–61.2	33
Discohexaster 2				
diameter	113	11	94–136	33
primary ray length	5.6	1.7	2.4–10.0	29
secondary ray length	51.7	4.8	43.4–60.7	29
Discohexaster 3				
diameter	75	15	44–94	22
primary ray length	7.1	1.7	3.8–12.1	22
secondary ray length	30.7	7.5	10.4–39.5	22
Oxyhexaster 1				
diameter	126	21	76–179	79
primary ray length	4.3	1.1	2.2–9.3	79
secondary ray length	58.8	10.3	32.0–85.3	79
Oxyhexaster 2				
diameter	74	25	31–116	23
primary ray length	5.9	1.1	4.3–8.0	23
secondary ray length	30.5	11.7	12.4–51.5	23
Oxyhexactin diameter	138		119–149	4

Microscleres (Fig. 11; Table 5) are three types of discohexasters and two types of oxyhexasters and their variants, rare hemioxyhexasters and oxyhexactins. The centrum in all categories tends to be varyingly swollen to the extent that the spicules approach asteroid forms. The oxyhexasters are extremely difficult to separate into types due to presence of intermediates. Discohexasters 1 (Fig. 11F) are semi-stellate with very short smooth primary rays, each supporting 4–8 gently curved secondary rays ornamented with reclined spines; terminal discs invariably have eight stout marginal teeth. Discohexasters 2 (Fig. 11G) are very slightly smaller spherical forms with each smooth primary ray supporting 2–4 straight thick terminal rays ornamented with reclined spines; the terminal discs have 5–9 marginal teeth. Discohexasters 3 (Fig. 11H) are small semi-stellate forms like discohexasters 1, with very short, smooth primary rays, each supporting 7–11 thin, curved, rough secondary rays, each ending in a terminal disc with 4–9 marginal teeth. Oxyhexasters 1, including hemioxyhexasters (Fig. 11H), are stout spherical forms with each short smooth primary ray supporting 1–3 secondary rays ornamented with dense reclined spines and ending in sharp tips. Oxyhexasters 2 (no SEM image available) are forms with very thin secondary rays and often broken; they have diverse characters but are not considered immature forms of oxyhexasters 1. The centres are very small or swollen to globular form and the distal ends of the primary rays are either very thin or globular, each primary end supports two or three thin secondary rays that appear totally smooth. We are not confident in recognising this as a spicule category since the only practical character in defining it is the thinness of the secondary rays and size. Oxyhexactins (no SEM available) are rare: only four have been verified in spicule surveys. They have characteristics of oxyhexasters 1 in the stoutness and ornamentation of their rays.

###### Etymology.

Named for the large, irregularly thorned hypodermal pentactins, that project from the body of this species (*variospinosum*, with irregular thorns; Latin).

###### Remarks.

The characters of these new specimens agree with the revised diagnosis of *Scyphidium* (see above) but differ from those of all eight recognised species of that genus. None has raised, thorned, hypodermal pentactins as prostalia. Only three species, *S.tuberculatum* (Okada, 1932), *S.jamatai* Tabachnick, 1991, and *S.australiense* Tabachnick, Janussen & Menschenina, 2008 (see above) have dermalia as mostly pentactins and atrialia as mostly hexactins, in agreement with the two new specimens. The sizes of the discohexasters are considerably smaller in all three than those in the new forms described here. On the basis of these and other differences, we are confident that the two new specimens described here represent a new species, here designated as *Scyphidiumvariospinosum* sp. nov.

##### Lanuginellinae

Taxon classificationAnimaliaLyssacinosidaRossellidae

Gray, 1872

32450BE3-C84B-577F-87E3-509BD36F73C9

###### Diagnosis.

Basiphytous, rarely lophophytous, often pedunculate, Rossellidae; dermalia hexactins, pentactins, stauractins, or diactins supported by large hypodermal pentactins; choanosomal spicules diactins, often supplemented by significant amount of hexactins; atrialia pentactins or hexactins often supported by large hypoatrial pentactins; dermal and atrial hexactins and pentactins frequently pinular; prostalia, if present, pentactins or diactins; microscleres include strobiloplumicomes, which may be absent in some species, oxy-, onycho-, or disco-tipped forms (hexasters, hemihexasters, hexactins); microdiscohexasters absent (after Dohrmann et al. 2017).

##### Caulophacus

Taxon classificationAnimaliaLyssacinosidaRossellidae

Schulze, 1886

E56C0B5C-3446-57F2-A58D-6ECCD0A015F4

###### Diagnosis.

Body is fungus-like or cup-like, basiphytose with long stalk. Choanosomal spicules are diactins and hexactins. Dermalia are pinular hexactins and/or pentactins. Atrialia are pinular hexactins and/or pentactins. Hypodermalia and hypoatrialia are pentactins. Microscleres are spicules of hexactinous or hexasterous forms with discoidal, onychoidal, and oxyoidal termination (emended from Janussen et al. 2004).

###### Type species.

Caulophacus (Caulophacus) elegans Schulze, 1886.

##### Caulophacus (Caulophacus)

Taxon classificationAnimaliaLyssacinosidaRossellidae

Schulze, 1886

0506246A-0399-5101-BC4F-A401B11BFCE9

###### Diagnosis.

Body is mushroom-shaped or cup-like, basiphytous with long stalk. Choanosomal spicules are diactins and hexactins. Dermalia and atrialia are pinular hexactins and/or pinular pentactins. Hypodermalia and hypoatrialia are pentactins. Microscleres are represented chiefly by spicules with discoidal terminations. They usually can be divided into two categories. The first are spicules with thick rays covered with dense spines: usually discohexactins but also discohexasters, hemidiscohexasters, and rarely discasters. The second are discohexasters with thin, smooth, or rough secondary rays usually in the form of lophodiscohexasters but sometimes calycocomes and spherical discohexasters are present among them (emended from Tabachnick 2002).

###### Remarks.

The subgenus Caulophacus is likely paraphyletic (Dohrmann 2019; MD, unpubl. results) and retained here for historical reasons only. Diagnoses of genus *Caulophacus* and subgenus Caulophacus are emended to include the new species Caulophacus (Caulophacus) serpens sp. nov. (described below) with mostly pinular pentactins as both dermalia and atrialia.

###### Type species.

Caulophacus (Caulophacus) elegans Schulze, 1886.

##### Caulophacus (Caulophacus) discohexaster

Taxon classificationAnimaliaLyssacinosidaRossellidae

Tabachnick & Lévi, 2004

07CBE753-0CA4-5689-93E2-515A627881FA

**Figure 12.**
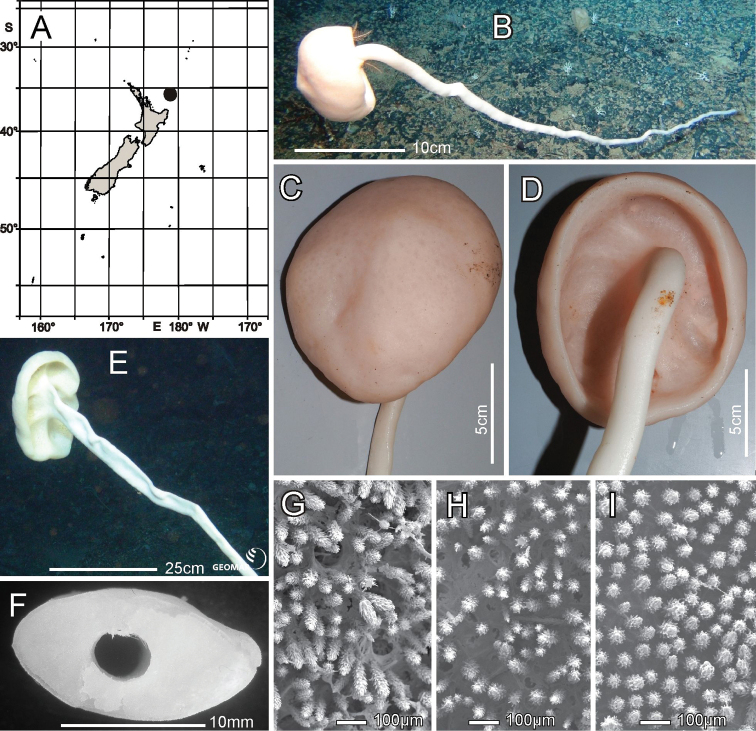
Caulophacus (Caulophacus) discohexaster Tabachnick & Lévi, 2004, NIWA 126342, 126343, distribution, skeleton, and morphology **A** distribution in New Zealand waters **B** smaller specimen, NIWA 126342 in situ **C** smaller specimen atrial surface on-deck image **D** smaller specimen dermal surface and stalk connection (deck images by PJS) **E** larger specimen, NIWA 126343 in situ **F** section of the stalk of the smaller specimen **G–I**SEM images of surfaces of smaller specimen; dermal **G** atrial **H** and stalk **I** at same magnification. Images **B** and **E** captured by ROV Team GEOMAR, ROV Kiel 6000 onboard RV Sonne (voyage SO254), courtesy of Project PoribacNewZ, GEOMAR and ICBM.

**Figure 13.**
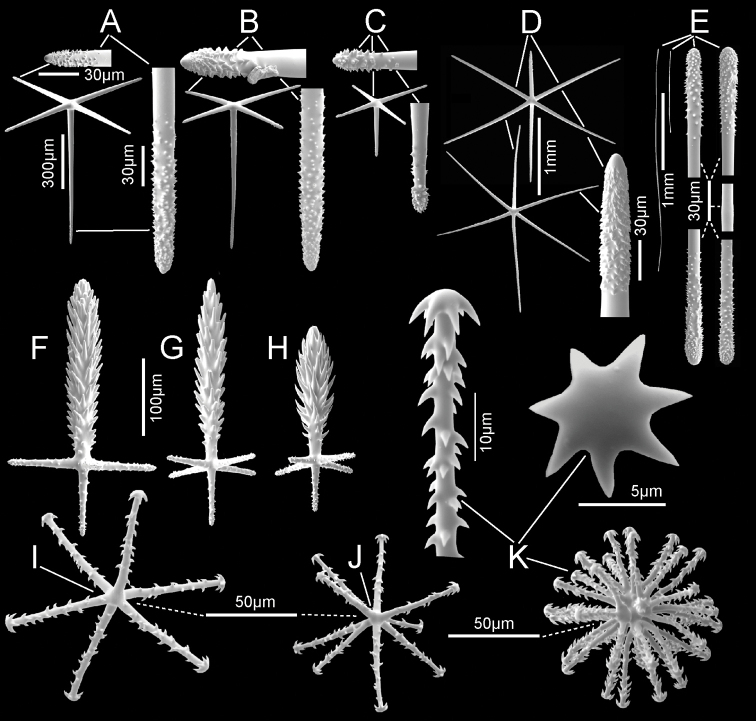
Caulophacus (Caulophacus) discohexaster Tabachnick & Lévi, 2004, NIWA 126342, spicules: **A** hypodermal body pentactin; whole and enlarged tangential and proximal ray ends **B** hypoatrial body pentactin; whole and enlarged tangential and proximal ray ends **C** hypodermal stalk pentactin; whole and enlarged tangential and proximal ray ends **D** choanosomal hexactins; two whole and enlarged ray end **E** choanosomal diactins; two whole and four enlarged ends and one central swelling **F** dermal body pinular hexactin **G** atrial body pinular hexactin **H** stalk dermal pinular hexactin **I** discohexactin **J** hemidiscohexaster **K**discohexaster and magnified terminal ray and terminal disc. Scale bars in **A** apply to **B** and **C**; scale bar is the same for **F–H, I–K**.

Figs 12 , 13 ; Table 6

###### Type and locality (not examined).

Holotype – MNHN HCL519, Norfolk Ridge, HALlPRO 2, Zoneco Stn BT 062, 24.71°S, 168.648°E, 720–1048 m.

###### Material examined.

NIWA 126342, RV Sonne Stn SO254/85ROV19_BIOBOX6, Southern Kermadec Ridge, 35.609°S, 178.854°E, 1163.6 m, 24 Feb 2016; NIWA 126343, RV Sonne Stn SO254/85ROV19_BIOBOX17, Southern Kermadec Ridge, 35.612°S, 178.852°E, 1149.8 m, 24 Feb 2017.

###### Distribution.

Known from the type locality, Norfolk Ridge near New Caledonia, and southern Kermadec Ridge, ~ 223 km N of East Cape, North Island, New Zealand.

###### Habitat.

Attached to hard substratum; depth 720 to 1348 m (New Zealand locations, Fig. 12A).

###### Description.

This description refers to New Zealand specimens only. Body forms a solitary mushroom cap-shaped upper body on a long, kinked, somewhat crooked, flattened, hollow stalk (Fig. 12B, E). Surfaces of the upper body are smooth (Fig. 12C, D) with a slight blunt eminence on the outer atrial face opposite the stalk insertion, but there is no indication of an osculum. The upper dermal surface lacks any visual indication of a lattice. The lower dermal surface of the specimen is divided by ridges into six depressions not seen in the smaller specimen. The stalks of both are flattened and that of the smaller bears a cylindrical central canal (Fig. 12F). SEM views of the dried surfaces show pores in the dermal body surface membrane (Fig. 12G), apparently contracted pores in the atrial body surface involving the atrial pinules (Fig. 12H), and no indication of pores on the dermal stalk surface (Fig. 12I). Dimensions of the smaller specimen upper body is ~ 12.5 × 10.6 cm in diameter; stalk is 15.9 × 9.0 mm in diameter and stalk canal is 4.1 × 3.3 mm in diameter. Length of the stalk could not be approximated. The larger specimen upper body is 25.0 × 15.9 cm in diameter while the flattened stalk is 5.9 cm wide. Measurable ostia of the smaller specimen (Fig. 12G) are 70 and 99 µm in diameter. Texture of the body is firm but compressible; the stalk is hard. Surface of all parts are smooth, consisting of tight palisades of pinular rays of dermalia and atralia supported on hypodermal and hypoatrial pentactins. There are no projecting prostalia. Colour in life is pale pinkish brown as are the specimens preserved in ethanol.

***Skeleton*.** Choanosomal skeleton of the body consists of a tight network of choanosomal hexactins and diactins. There is no evidence of fusion between any spicules within the body. Microscleres are scattered evenly throughout the choanosome. The stalk internal skeleton is composed of large diactins oriented longitudinally and fused by synapticula. Ectosomal skeleton of the dermal and atrial sides consists of tightly packed pinular hexactins and very few pinular pentactins (1.6% of 623 assessed). These are supported on, respectively, hypodermal and hypoatrial pentactins which are never raised above the surfaces. Microscleres are present as in the choanosome.

***Spicules*.** Megascleres (Fig. 13; Table 6) are hypodermal pentactins, choanosomal hexactins and diactins, and pinular hexactins and a few pentactins. Hypodermal pentactins of the body (Fig. 13A) are regular and usually smooth except for spined ray ends; 8% have indistinct spines on the proximal part of the proximal ray. The proximal rays are longer, averaging 1.24 × the length of tangential rays. Hypoatrial pentactins of the body (Fig. 13B) are regular and spined on both ray-ends and 61% of them on the proximal part of the proximal rays. The proximal ray is longer, averaging 1.62 × the length of tangential rays. Hypodermal pentactins of the stalk (Fig. 13C) are regular in shape but significantly smaller than those of the body; they are spined only on ray ends. Tangential and proximal rays are approximately equal in length. Choanosomal hexactins (Fig. 13D) are restricted to the body; rays are smooth, and spines are restricted to the ray ends except where the ray is exceptionally short. These hexactins occur in two forms, one with a short spiny ray (upper figure) and the other with all rays approximately equal in length (lower figure). Choanosomal diactins (Fig. 13E) are straight or slightly curved and are smooth except for ends on which they have small but detectable central swellings. Dermal pinular hexactins of the body (Fig. 13F) have bushy, nearly cylindrical pinular rays with a short, thick, rounded apical tip. Tangential and proximal rays are entirely spined and approximately similar in size and shape. Very rarely, these and pinules of other body surfaces are pentactine with only a round nub in place of the proximal ray. Atrial pinular hexactins of the body (Fig. 13G) have pinular rays that taper in length of scales at both ray ends, resulting in fusiform shape. The pinular ray has a thick and rounded tip. Tangential and proximal rays are entirely spined and similar in size and shape. Stalk pinular hexactins (Fig. 12H) have a pinular ray that is squat and slightly wider than those of the body spicules. Scale lengths taper basally and apically and again the apex of the pinule is a blunt, thick cap. Tangential and proximal rays are entirely spined and similar in size and shape.

**Table 6.** Spicule dimensions (µm) of Caulophacus (Caulophacus) discohexaster Tabachnick & Lévi, 2004, NIWA 126342.

Parameter	mean	s.d.	range	no.
Hypodermal body pentactin				
tangential ray length	484	83	235–637	76
tangential ray width	33.9	5.3	13.0–43.4	79
proximal ray length	602	214	256–967	56
proximal ray width	38.5	5.9	18.0–52.0	72
Hypoatrial body pentactin				
tangential ray length	495	81	318–668	61
tangential ray width	31.3	5.2	18.3–41.3	62
proximal ray length (mm)	804	173	213–1088	57
proximal ray width	35.8	6.3	6.8–50.5	60
Hypodermal stalk pentactin				
tangential ray length (mm)	289	46	174–415	100
tangential ray width	22.7	3.5	14.0–32.0	64
proximal ray length (mm)	296	32	205–355	43
proximal ray width	25.4	3.8	14.7–33.3	50
Choanosomal hexactin ray				
length (mm)	1.1	0.5	0.5–2.1	53
width	48.4	13.7	17.6–78.5	53
Choanosomal diactin				
length (mm)	1.6	0.5	0.7–2.9	52
width	7.7	2.1	4.6–13.3	52
Body dermal pinular hexactin				
pinular ray length	200	28	158–241	52
basal ray width	16.6	2.2	11.3–21.4	51
maximum ray width	49.7	6.2	36.6–70.6	52
tangential ray length	84.0	18.5	51.8–115.1	52
ray width	12.5	2.0	8.6–17.1	52
proximal ray length	80.9	14.1	47.7–111.5	50
ray width	12.4	2.3	8.8–17.3	52
Body atrial pinular hexactin				
pinular ray length	242	17	180–264	51
basal ray width	14.7	1.6	11.3–17.9	51
maximum ray width	49.9	5.7	37.0–61.4	51
tangential ray length	71.2	9.5	53.2–100.0	51
ray width	10.7	1.6	7.514.3	51
proximal ray length	71.2	8.1	39.9–88.5	51
ray width	10.4	1.6	7.6–14.9	51
Stalk dermal pinular hexactin				
pinular ray length	170	15	131–198	51
basal ray width	16.2	1.9	11.2–20.6	51
maximum ray width	54.8	5.8	43.9–69.2	51
tangential ray length	59.1	8.4	7.5–15.8	51
ray width	12.0	1.9	7.5–15.8	51
proximal ray length	63.8	6.5	47.9–88.7	48
ray width	11.4	1.6	8.4–14.6	51
Discohexactin				
diameter	131	21	75–163	51
ray width	4.7	1.1	2.5–7.6	51
Hemidiscohexaster				
diameter	119	14	90–150	52
primary ray length	7.3	1.3	4.0–10.0	51
secondary ray length	52.7	7.2	34.4–69.9	52
Discohexaster				
diameter	108	18	55–143	52
primary ray length	7.0	1.7	3.1–10.7	51
secondary ray length	47.4	7.8	22.4–60.3	52

Microscleres (Fig. 13; Table 6) are discohexactins (43% of 221 assessed), hemidiscohexactins (54.7%), and discohexasters (2.3%); all are thick-rayed forms. Discohexactins (Fig. 13I) have rays ornamented with large, reclined spines and a terminal disc with 5 (4–7) marginal teeth. Hemidiscohexasters (Fig. 13J) are similar with at least one ray being branched and at least one ray being unbranched; the total number of rays is 9.4 (7–13). Terminal discs are similar to discohexactins. Discohexasters (Fig. 13K) have all primary rays branched varying from 2–6 terminal rays on each primary ray. Terminal discs have 3–8 marginal teeth.

###### Remarks.

The morphological characters of the two New Zealand specimens place them clearly in subgenus Caulophacus (Caulophacus), of which there are 22 recognised species. Table 7 compares the key morphological characters that differentiate them from each other and from the New Zealand specimens, NIWA 126342 and NIWA 126343. We admit that some of these differences are subjective and the list is not exhaustive. Table 7 shows only a single species, C. (C.) discohexaster, that has a single morphological difference from the specimens described here, i.e., the visual impression of the dermal pinule (Tabachnick and Lévi 2004: 51, fig. 24A). Measurement of the dermal pinule in that figure shows that the figured pinular ray is too wide (84 µm) to fit within the data given for the pinular ray of the dermal hexactin given there (106–220 µm/30–46 µm). Removing this illustration error from the list results in no differences between the New Zealand specimens and the type material described by Tabachnick and Lévi (2004) from New Caledonia; therefore, we assign the specimens to that species.

**Table 7.** Comparison of the key morphological characters that differentiate the 24 species of Caulophacus (Caulophacus) from each other and from New Zealand specimens of Caulophacus (Caulophacus) discohexaster Tabachnick & Lévi, 2004) (NIWA 126342 and NIWA 126343).

taxa	location	body shape differs radically	dermal pinule differs visually in shape	atrial pinule differs visually in shape	stalk pinule differs visually in shape	dermal pinule, 2 or more forms	dermal, atrial or stalk pinules mostly pentactins	dermal, atrial or stalk pinules exclusively pentactins	dermal pinule ray is much shorter	atrial pinule ray is much shorter	dermal and atrial pinules, basal rays of nearly smooth	choanosomal hexactins, some or all are centrally spined	hypodermal pentactins, some or all are centrally spined	hypodermal pentactins are very small	hypodermal pentactins of stalk, atrial pinules are mostly pentactins	hypodermal pentactins of stalk, some or all are centrally spined	hypoatrial pentactins tangential rays, some or all centrally spined	hypoatrial pentactins very small	microsclere hemidiscohexasters absent	microsclere discohexasters absent	microsclere discohexactins absent	microsclere discohexasters include thin-rayed forms	microsclere discohexasters in two or more forms	microsclere oxy-tipped forms present
* abyssalis *	Argentine Basin		x			x						x	x						x					
* adakensis *	Aleutian Islands											x	x				x							
* agassizi *	Gulf of Maine/Bay of Fundy		x	x			x					x	x				x		x					
* antarcticus *	East Antarctic Wilkes Land		x	x			x							x				x		x				
* arcticus *	Greenland and Arctic Ocean		x	x					x				x		x		x					x		
* basispinosus *	Indian Ocean						x					x	x				x		x	x				x
* chilense *	Central Chile	x										x	x				x					x		x
* cyanae *	Mexican Tropical Pacific		x	x			x						x						x				x	x
* discohexactinus *	Antarctica						x						x						x			x	x	
* discohexaster *	New Caledonia		x																					
* elegans *	North Pacific		x	x							x		x											
* galatheae *	Indian Ocean								x				x						x	x				x
* hadalis *	Southern Kermadec Ridge		x				x									x			x					
* instabilis *	South Orkney Islands				x								x						x					
* latus *	Crozet Islands		x	x					x				x									x		x
* oviformis *	East Antarctica	x	x	x							x	x							x		x			
* palmeri *	Drake Passage									x									x		x			
* pipetta *	East Antarctica	x	x	x							x		x									x	x	
* ramosus *	Kermadec Trench							x							x									
* schulzei *	Panama Bight, Pacific Ocean				x						x		x				x					x	x	
* serpens *	Kermadec Trench						x																	
* scotiae *	Weddell Sea, Antarctica		x	x									x									x	x	
* variens *	Western Pacific Ocean		x	x								x							x			x	x	
* wilsoni *	Eastern Pacific Ocean	x	x	x									x						x			x		

*Taxonomic authorities have been excluded from this column but are available from van Soest et al. (2021)

##### Caulophacus (Caulophacus) serpens

Taxon classificationAnimaliaLyssacinosidaRossellidae

Reiswig, Dohrmann & Kelly sp. nov.

F8338B31-A2F8-591C-87DA-A811AB9862BB

http://zoobank.org/BF258FB6-7C4B-4A90-95B9-5E8BF02B8B5A

**Figure 14.**
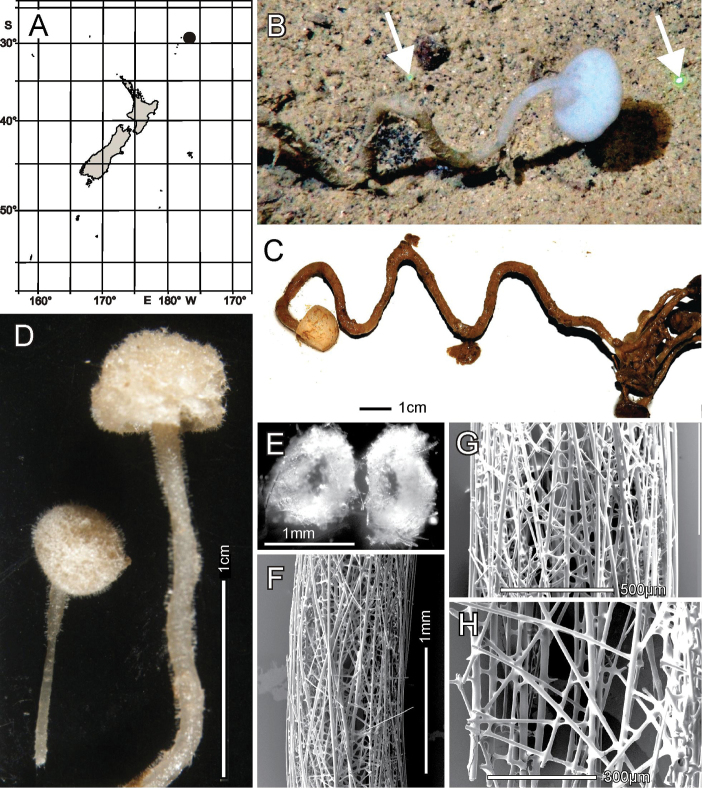
Caulophacus (Caulophacus) serpens sp. nov., NIWA 126084, distribution, skeleton, and morphology **A** distribution in New Zealand waters, collection location of holotype on the Kermadec Trench slope **B** in situ image of the largest specimen body and the irregular undulating stalk associated with it. The laser spots indicated by the arrows are 6.24 cm apart **C** deck image of the same with smaller specimens in the stalk tangle at right (image by PJS) **D** two smaller bodies and their stalks, previously attached to the main mass, used for spicule analysis **E** cross sections of the uncleaned stalk of the larger specimen in **D**; **F** Acid-cleaned part of the stalk of the same (SEM) **G** closer view of the outer stalk surface showing most fused diactins oriented nearly parallel to the stalk axis (SEM) **H** close view of the internal stalk surface showing most superficial spicules oriented at large angles to the stalk axis (SEM). Image **B** captured by ROV Team GEOMAR, ROV Kiel 6000 onboard RV Sonne (voyage SO254), courtesy of Project PoribacNewZ, GEOMAR, and ICBM.

**Figure 15.**
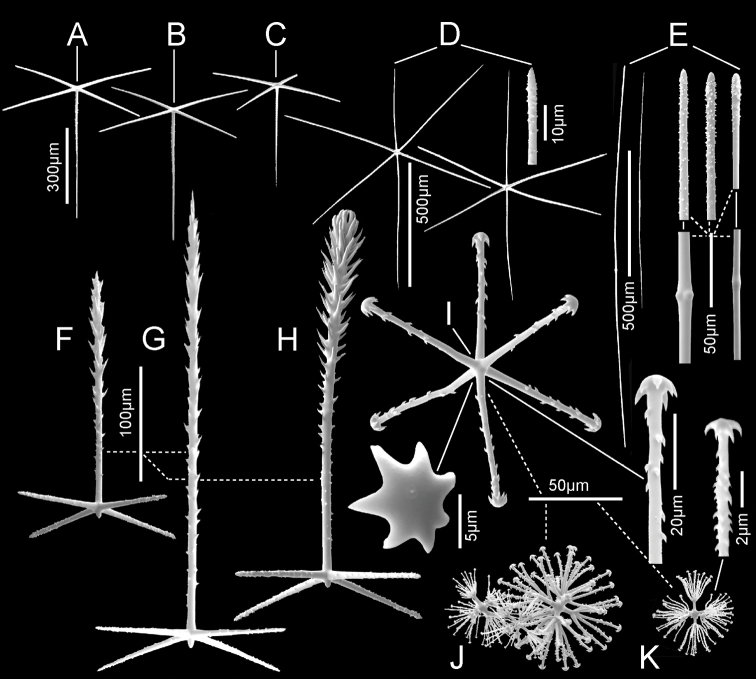
Caulophacus (Caulophacus) serpens sp. nov., NIWA 126084, spicules **A** hypodermal body pentactin **B** hypoatrial body pentactin **C** hypodermal stalk pentactin **D** two whole choanosomal hexactins and an enlarged ray end **E** two whole choanosomal diactins and enlarged ends and central swellings; **F** dermal body pinular pentactin **G** atrial body pinular pentactin **H** stalk dermal pinular pentactin **I** discohexactin with enlarged terminal ray and facial view of end disc **J** large thick-rayed discohexaster with two smaller thin-rayed discohexasters entangled **K** thin-rayed discohexaster and magnified terminal ray. Scale bar in **A** applies to **B–E** scale bar in **F** applies to **G** and **H** whole microscleres are at the same scale.

Figs 14 , 15 ; Table 8

###### Material examined.

***Holotype***NIWA 126084, RV Sonne Stn SO254/22ROV06_BIOBOX6, Kermadec Trench slope, 29.266°S, 176.702°W, 4816 m, 04 Feb 2017.

###### Distribution.

Known only from the type locality, the Kermadec Trench slope, north of New Zealand (Fig. 14A).

###### Habitat.

Attached to large pieces of rubble lying on a sediment plain at 4816 m.

###### Description.

Morphology of the holotype a rhizome-like, hard, hollow, thin stem that creeps across the sediment seabed, attaching to rubble here and there, in places forming a tangled mass, from which arises the main mushroom-shaped body on a zigzag stem (Fig. 14B, C), and two tiny, mushroom-shaped bodies (Fig. 14D). Overall dimension of the holotype, spreading across the seabed, is 64 cm. Dimension of the larger body (Fig. 14B) is 22.2 mm in diameter and 11.2 mm in height; its associated stalk is 3.6 (2–6–4.3) (n = 12) mm in diameter. The associated stalk measures 3.3 (2.7–4.0) (n = 29) mm in diameter, the length, measured in conformation from the body to the stalk tangle, is 167 mm. The smaller bodies (Fig. 14D) are 7.2 × 5.4 and 5.2 × 4.3 mm in diameter and height, respectively; the stalk of the larger one is 1.1 (1.0–1.3) (n = 12) mm in diameter. The piece of thick stalk received from NIWA is 72 mm long and 3.0 (2.8–3.3) (n = 12) mm in diameter, approximately the same gauge as the convoluted stalk shown in the in situ and deck images. Surfaces of the body are a bit lumpy and fuzzy (Fig. 14D); that of the thin attached stalks is also fuzzy. There are no projecting prostal spicules. Colour of the body in life is white, and the stem pale brown; when preserved in ethanol it is very pale brown, almost white.

***Skeleton*.** Choanosomal skeleton of the body is a network of diactins and hexactins. There is no evidence of fusion between any spicules within the body. Spicule fusion is restricted to the choanosomal diactins of the hollow stalks where the diactins are joined by synapticula and points of spot contacts between spicules. Microscleres are scattered evenly throughout the choanosome. Ectosomal skeleton of the dermal and atrial sides of the body and living stalks consists of tightly packed pinular pentactins and very few pinular hexactins (1.3% of 374 assessed). These are supported on, respectively, hypodermal and hypoatrial pentactins which are never raised above the surfaces. Microscleres are present as in the choanosome.

***Spicules*.** Megascleres (Fig. 15; Table 8) are hypodermal and hypoatrial pentactins, choanosomal hexactins and diactins, and pinular pentactins and a few pinular hexactins. Hypodermal pentactins of the body (Fig. 15A) are regular and usually smooth except for spined ray ends; 31% have macrospines on the central part of the proximal ray. The proximal rays are longer, averaging 1.21 × the length of tangential rays. Hypoatrial pentactins of the body (Fig. 15B) are regular and spined on both tangential and proximal ray ends; macrospines are present on the central part of most (60%) proximal rays but all tangential rays lack macrospines. The proximal ray is longer, on average 1.86 × the length of tangential rays. Hypodermal pentactins of the stalk (Fig. 15C) are regular in shape but significantly smaller than those of the body; they are spined on ray ends but macrospines are uncommon (12%) on the central part of only the proximal rays. Proximal rays are generally longer, on average 1.49 × tangential ray length. Choanosomal hexactins (Fig. 15D) are restricted to the body; rays are smooth, and spines are restricted to the ray ends. Macrospines are never found in the central part of these spicules. Choanosomal diactins (Fig. 15E) are straight or slightly curved and are smooth except for ends; they have small but detectable central swellings. Dermal pinular pentactins of the body (Fig. 15F) have narrow pinular rays topped with a short, sharp apical spine. Their basal rays are entirely spined and end in abruptly pointed tips. Approximately 10% of the dermal pinules are hexactine forms. Atrial pinular pentactins of the body (Fig. 15G) have narrow pinular rays like the dermal pinules but with a longer pinular ray (on average 2.1 ×) and longer apical spine; basal rays are like those of the dermal pinules. Stalk pinular hexactins (Fig. 15H) have a pinular ray that is narrow in its basal half but curves to one side and swells in width apically, assuming an overt club-shape. It has no atrial spine since the apex is enfolded by the apical scales. Basal rays are like those of the dermal and atrial pinules.

**Table 8.** Spicule dimensions (µm) of Caulophacus (Caulophacus) serpens sp. nov., NIWA 126084.

Parameter	mean	s.d.	range	no.
Hypodermal body pentactin				
tangential ray length	398	187	192–1385	43
tangential ray width	13.5	2.7	8.2–19.6	41
proximal ray length	482	207	210–865	30
Hypoatrial body pentactin				
tangential ray length	360	72	206–689	46
tangential ray width	14.7	3.0	8.0–20.1	47
proximal ray length	670	96	450–898	46
Hypodermal stalk pentactin				
tangential ray length	205	64	117–460	51
tangential ray width	10.8	2.2	7.2–19.0	50
proximal ray length	305	129	140–789	49
Choanosomal hexactin ray				
length	555	75	286–718	53
width	10.3	2.2	5.5–14.2	58
Choanosomal diactin				
length (mm)	1.31	0.35	0.63–2.22	50
width	7.1	1.4	4.9–11.4	50
Body dermal pinular pentactin				
pinular ray length	218	28	164–273	29
basal ray width	8.4	1.6	6.0–14.6	44
maximum ray width	21.3	3.0	14.7–29.1	60
tangential ray length	95.5	12.5	72.2–117.8	30
ray width	7.0	1.0	4.8–9.3	53
Body dermal pinular hextactin				
pinular ray length	169	14	156–184	3
pinular ray basal width	7.7	0.9	6.2–8.8	7
pinular ray maximum width	25.2	4.2	21.7–30.6	4
tangential ray length	93.5	15.0	84.5–110.8	3
tangential ray width	6.5	1.0	4.8–7.7	7
proximal ray length	77.1	13.1	63.8–90.0	3
proximal ray width	6.8	0.6	6.2–7.7	4
Body atrial pinular pentactin				
pinular ray length	362	51	263–440	30
pinular ray basal width	8.2	1.2	5.0–10.2	55
pinular ray maximum width	17.9	3.1	11.8–27.0	44
tangential ray length	123	16	98–167	44
tangential ray width	7.2	1.2	4.2–9.8	59
Stalk dermal pinular pentactin				
pinular ray length	283	22	242–326	30
pinular ray basal width	8.6	1.5	6.0–13.7	50
pinular ray maximum width	32.3	6.3	18.2–47.9	50
tangential ray length	102	14	65–133	34
tangential ray width	7.0	1.1	4.5–9.3	57
Discohexactin				
diameter	185	18	142–225	58
ray width	4.6	0.7	2.9–6.8	58
Thick-ray discohexaster				
diameter	94	32	48–139	9
primary ray length	7.5	1.4	5.0–9.4	9
secondary ray length	39.8	15.8	18.0–60.7	9
Thin-ray discohexaster				
diameter	46.1	4.5	34.0–58.0	59
primary ray length	7.6	1.2	4.4–11.4	59
secondary ray length	15.4	2.0	8.8–21.3	59

Microscleres (Fig. 15; Table 8) are discohexactins, thick-ray discohexasters and thin-ray discohexasters. Discohexactins (Fig. 15I) are the most abundant microscleres; they have rays ornamented with large, reclined spines and a terminal disc with 5–8 marginal teeth. Thick-ray discohexasters (Fig. 15J) are the least abundant microsclere; they are spherical, have 6–9 thorned terminal rays on each smooth primary ray, and terminal discs have 4–8 marginal teeth. Thin-ray discohexasters (Fig. 15J, K) are among the most numerous microscleres, but comparing their abundance with discohexactins is not possible since detection of the two spicule types requires different microscope arrangements. They are semi-stellate with each smooth primary ray supporting 16 (8–28) (n = 16) thorned terminal rays ending in discs with 3–7 marginal teeth.

###### Etymology.

Named for the rhizome-like stem that may form tangled, convoluted stems from which the main bodies arise, the whole creeping along the substrate (*serpens*, creeping; Latin).

###### Remarks.

The morphological character of all microscleres being discoid, places this species in the subgenus Caulophacus (Caulophacus). In comparing them to the 22 recognised species of this subgenus (Table 7), it is apparent that there are no forms known with both dermal and atrial spicules as mainly pinular pentactins. It is thus clear that the form described here is the holotype of a new species named Caulophacus (Caulophacus) serpens sp. nov.

##### Caulophacus (Caulophacus) ramosus

Taxon classificationAnimaliaLyssacinosidaRossellidae

Reiswig, Dohrmann & Kelly sp. nov.

CAA72752-CEEB-5252-9626-61AFC687BFC1

http://zoobank.org/C3DFB4B3-84C0-4794-A26F-84CB58A9F7E3

**Figure 16.**
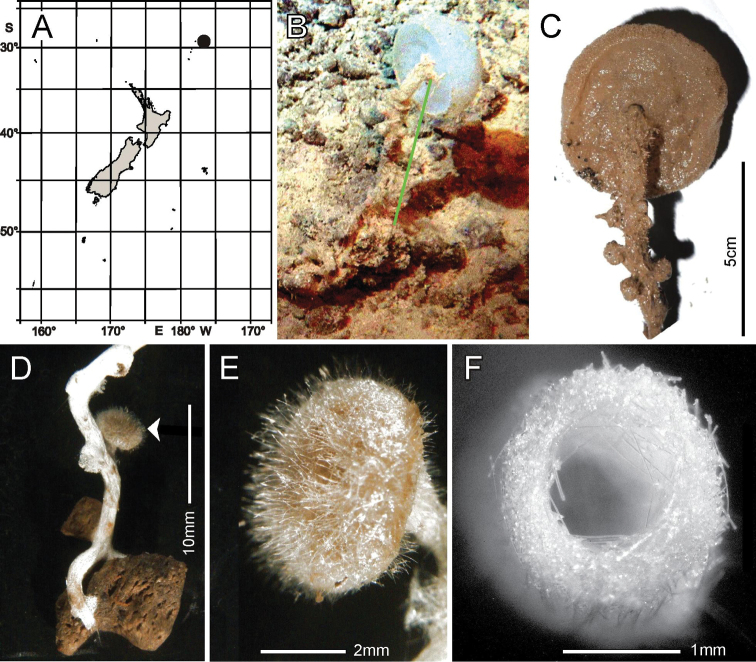
Caulophacus (Caulophacus) ramosus sp. nov., NIWA 126085, distribution, skeleton, and morphology **A** map of collection location on Kermadec Trench slope **B** in-situ image of the largest body; the irregular undulating stalk associated with it is largely hidden in accumulated sediment. The green line is 6.24 cm long copied from between laser spots elsewhere in the image **C** deck image of the large body, which was unavailable for taxonomic description since used for other analyses (image by PJS) **D** a smaller body (arrowhead) attached presumably to the same stalk but lower in the sinuous section with multiple branching attachments to small cobbles **E** The same body enlarged to show the plush of long atrial pinular pentactins **F** cross section of the larger contort white stalk and its central canal. Image **B** captured by ROV Team GEOMAR, ROV Kiel 6000 onboard RV Sonne (voyage SO254), courtesy of Project PoribacNewZ, GEOMAR, and ICBM.

**Figure 17.**
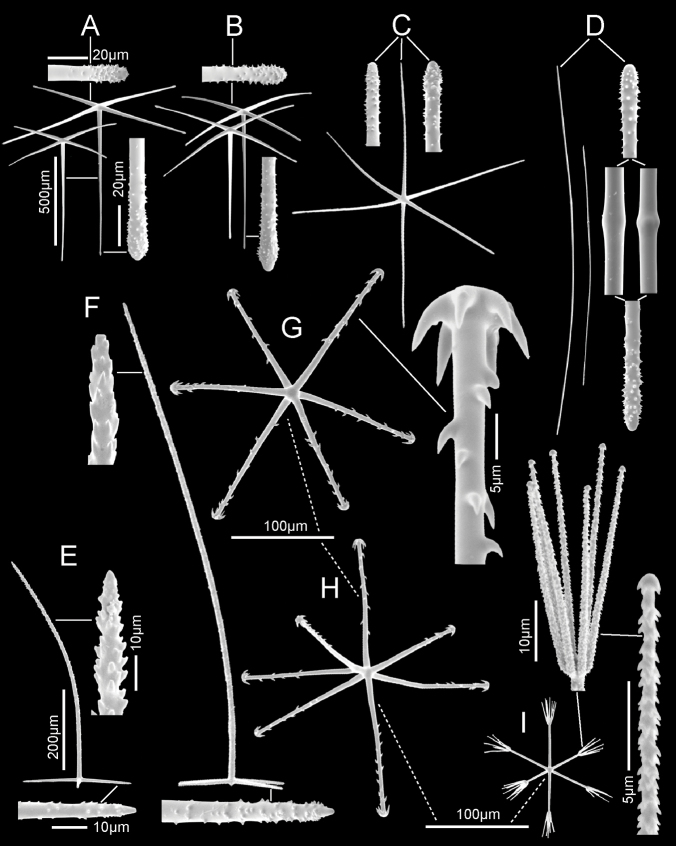
Caulophacus (Caulophacus) ramosus sp. nov., NIWA 126085, spicules **A** two whole hypodermal body pentactins and enlarged ray tips **B** two whole hypoatrial body pentactins and enlarged ray ends **C** a choanosomal hexactin and two enlarged ray tips **D** t wo whole choanosomal diactins and enlarged ends and central swellings **E** dermal body pinular pentactin and enlarged ray ends **F** atrial body pinular pentactin and enlarged ray ends **G** discohexactin and enlarged ray end **H** hemidiscohexaster **I** thin-rayed stellate discohexaster with enlarged secondary ray tuft and a secondary ray. Scale bars in **A** apply to **B–D** scales in **E** apply to **F** whole microscleres are at the same scale.

Figs 16 , 17 ; Table 9

###### Material examined.

***Holotype***NIWA 126085, RV Sonne Stn SO254/22ROV06_BIOBOX4, Kermadec Trench slope, 29.266°S, 176.702°W, 4819 m, 04 Feb 2017.

###### Distribution.

Known only from the type locality, the Kermadec Trench slope, north of New Zealand (Fig. 16A).

###### Habitat.

Attached to hard substratum; depth 4819 m.

###### Description.

Morphology of the holotype is a compound mass of a thin, convoluted stalk-part, with at least one small mushroom-shaped body branching from it (Fig. 16D, E), and a longer, thicker, upright stalk-part bearing a larger terminal mushroom-shaped body (Fig. 16B, C). The larger upright stalk has six lateral knobs just below the larger body on its stalk (Fig. 16C) whose nature and function are unknown, possibly sites for attachment to a hard substratum, or are new buds. The lower convoluted stalk part branches into many attachment points, at least eight within a 27 mm length (Fig. 16D). The smaller of the two bodies attached to this stalk system has a felt-like cover of long pinular pentactins on the outer surface (Fig. 16E); we have had no opportunity to examine the larger body. The stalk in all parts is hollow (Fig. 16F). Overall dimension of the larger body in the in-situ image is 45.5 mm in diameter with the stalk having a diameter of 5.9 mm at a point 5 mm below the attachment. The smaller specimen is 5.3 mm in diameter and 3.6 mm in height. Stalk diameter varies from 1.0 mm at the short branch joining the small specimen to the convoluted stalk which is mostly ca 1.8 mm thick. The connection of the convoluted part of the stalk to the thicker upright stalk part was not available for assessment. Surfaces of the small body are covered by a villous plush of long pinular pentactins, but there are no special prostalia present. The lower convoluted stalk surfaces appear devoid of any visible surface spicules, but spicule preparations of this apparently “barren” stalk still show that typical stalk spicules are present. Thus, spicules obtained from stalks may derive from other locations on the specimen and should be considered as possibly from other original sources. Surfaces of the upper straight stalk and the terminal larger body are known only from fresh seawater-wet lab photos; they are covered by a thick spiny layer of brown tissue (Fig. 16C). Colour of the body in life is translucent white; when preserved in ethanol it is pale brown.

***Skeleton*.** Choanosomal skeleton of the body is a network of diactins and hexactins. There is no evidence of fusion between any spicules within the body. Spicule fusion is restricted to the choanosomal diactins of the hollow stalks where the diactins are joined by fusion at spot contacts and by relatively long synapticula forming ladders. Microscleres are scattered evenly throughout the choanosome. Ectosomal skeleton of the dermal and atrial sides of the body consists of tightly packed pinular pentactins; no pinular hexactins are present. These are supported on, respectively, hypodermal and hypoatrial pentactins, which are never raised above the surfaces. Microscleres are present as in the choanosome.

***Spicules*.** Megascleres (Fig. 17; Table 9) are hypodermal and hypoatrial pentactins, choanosomal hexactins and diactins, and pinular pentactins. Hypodermal pentactins of the body (Fig. 17A) are regular and smooth except for spined ray ends. The proximal rays are longer, averaging 1.26 × the length of tangential rays. Hypoatrial pentactins of the body (Fig. 17B) are also regular and smooth except for spined areas on both tangential and proximal ray ends. The proximal ray is longer, averaging 1.41 × the length of tangential rays. Hypodermal pentactins of the stalk (not figured) are regular in shape but significantly smaller than those of the body. Choanosomal hexactins (Fig. 17C) are restricted to the body; rays are smooth and spines are present only on ray ends. Macrospines are never found in the central part of these spicules. Choanosomal diactins (Fig. 17D) are straight or slightly curved and are smooth except for the ends; they have small but detectable central swellings. Dermal pinular pentactins of the body (Fig. 17E) have narrow pinular rays topped with a short, blunt apical spine. Their basal rays are entirely spined and end in abruptly rounded tips. Atrial pinular pentactins of the body (Fig. 17F) have narrow pinular rays like the dermal pinules, but with a longer pinular ray (on average 1.25 ×); however, presence of an apical spine was not determined since all of these examined in SEM had broken tips. Basal rays are like those of the dermal pinules. Stalk pinular pentactins (not figured) are morphologically similar to the dermal body pentactins.

**Table 9.** Spicule dimensions (µm) of Caulophacus (Caulophacus) ramosus sp. nov., NIWA 126085.

Parameter	mean	s.d.	range	no.
Hypodermal body pentactin				
tangential ray length	417	107		42
tangential ray width	19.7	4.0		44
proximal ray length	526	187		35
Hypoatrial body pentactin				
tangential ray length	438	73		53
tangential ray width	21.2	2.7		54
proximal ray length	617	129		52
Hypodermal stalk pentactin				
tangential ray length	203	37		46
tangential ray width	10.8	2.5		49
proximal ray length	337	187		25
Choanosomal hexactin				
short ray length	497	70		14
long ray width	847	143		16
ray width	24.3	3.0		16
Choanosomal diactin				
length (mm)	1.7	0.7		44
width	8.6	2.3		44
Body dermal pinular pentactin				
pinular ray length	504	133		8
pinular ray basal width	9.2	2.1		29
pinular ray maximum width	11.2	3.0		29
tangential ray length	118	22		20
tangential ray width	7.4	1.7		30
Body atrial pinular pentactin				
pinular ray length	630	127		41
pinular ray basal width	9.2	1.6		40
pinular ray maximum width	11.7	1.8		41
tangential ray length	122	20		35
tangential ray width	7.6	1.5		40
Stalk dermal pinular pentactin				
pinular ray length	530	125		30
pinular ray basal width	11.4	4.9		30
pinular ray maximum width	16.4	7.0		30
tangential ray length	132	33		23
tangential ray width	8.8	3.9		30
Discohexactin				
diameter	263	30		52
ray width (from SEM only)	6.7	0.9		5
Thin-ray stellate discohexaster				
diameter	154	24		50
primary ray length	50.9	7.5		50
secondary ray length	26.2	7.8		50

Microscleres (Fig. 17; Table 9) are thick-rayed discohexactins and rare hemidiscohexasters and thin-rayed stellate discohexasters. Discohexactins (Fig. 17G) are the most abundant microscleres; they have rays ornamented with large, reclined spines and a terminal disc with 5–8 marginal teeth. Rare hemidiscohexasters (Fig. 17H) are similar to the discohexactins. Thin-rayed stellate discohexasters (Fig. 17I) have long, smooth primary rays supporting a narrow shorter brush of 3–9 straight, rough, terminal rays ending in small discs.

###### Etymology.

Named for the lower, convoluted stalk part, which branches into many attachment points (*ramosus*, branching; Latin).

###### Remarks.

The morphological character of all microscleres being discoid, places this species in the subgenus Caulophacus (Caulophacus). In comparing it to the 22 recognised species of this subgenus (Table 7), it is apparent that there are no forms known with all pinules, both dermal and atrial, as exclusively pentactins. It is very like the previous described new species, C. (Caulophacus) serpens sp. nov. in its mainly, but not exclusively, pinular pentactins, and in the body form with a significant portion of the stalk convoluted, attached by many attachment sites and compound bearing several bodies. The two differ, however, in pinule morphology and types of microscleres. Also, molecular data (MD, unpubl. results) suggest a closer relationship of this specimen to C. (Caulophacus) arcticus, C. (Caulodiscus) valdiviae, and C. (Oxydiscus) weddelli than to C. (C.) serpens sp. nov. Since it cannot be assigned to any of the former species on the basis of morphology, it is thus clear that the form described here represents the holotype of a new species named Caulophacus (Caulophacus) ramosus sp. nov.

## Conclusions

ith the material described herein, the known diversity of rossellids from the surrounding waters of New Zealand has almost doubled, from previously known nine species in five genera to 17 species in eight genera, including six species and one genus new to science:

*Bathydoruspoculum* Reiswig, Dohrmann & Kelly, sp. nov.

Caulophacus (Caulodiscus) onychohexactinus Tabachnick & Lévi, 2004

Caulophacus (Caulophacus) discohexaster Tabachnick & Lévi, 2004

Caulophacus (Caulophacus) hadalis Lévi, 1964

Caulophacus (Caulophacus) ramosus Reiswig, Dohrmann & Kelly, sp. nov.

Caulophacus (Caulophacus) schulzei Wilson, 1904

Caulophacus (Caulophacus) serpens Reiswig, Dohrmann & Kelly, sp. nov.

Crateromorpha (Aulochone) cylindrica (Schulze, 1886)

Crateromorpha (Aulochone) haliprum Tabachnick & Lévi, 2004

Crateromorpha (Caledochone) caledoniensis Tabachnick & Lévi in Tabachnick (2002)

*Nubespoculiformis* Reiswig, Dohrmann & Kelly, gen. nov., sp. nov.

*Nubestubulata* Reiswig, Dohrmann & Kelly, gen. nov., sp. nov.

*Rossellaijimai* Dendy, 1924

*Scyphidiumaustraliense* Tabachnick, Janussen & Menschenina, 2008

*Scyphidiumvariospinosum* Reiswig, Dohrmann & Kelly, sp. nov.

*Sympagellaclavipinula* Tabachnick & Lévi, 2004

*Symplectellarowi* Dendy, 1924

Descriptions of two further new rossellids (Lanuginellinae) from RV Sonne cruise SO254 could not be completed by HMR and will be reported elsewhere, together with numerous other hexactinellid specimens collected on that cruise.

## Obituary

**Figure 18.**
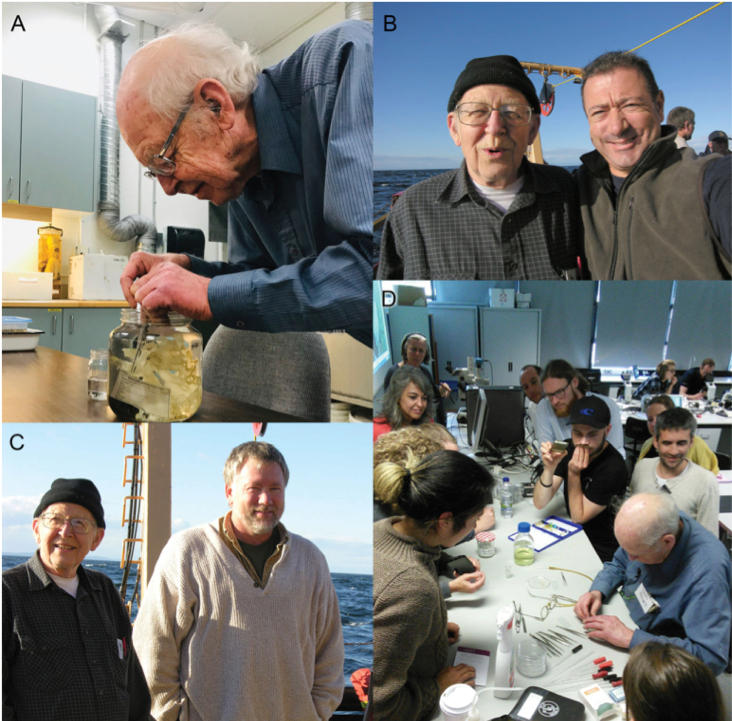
Dr Henry Michael Reiswig (8 July 1936–4 July 2020) of Saanichton, British Columbia, Canada **A** henry at work examining a dictyonal glass sponge. Image provided by Heidi Gartner, Invertebrate Collection Manager and Researcher at the Royal British Columbia Museum, with permission **B** henry and Dr Manuel Maldonado, Sponge Ecobiology and Biotechnology Group, Department of Marine Ecology, Centro de Estudios Avanzados de Blanes (CEAB), onboard the Canadian Coast Guard Ship Vector in the Strait of Georgia, Vancouver, using the ROV Ropos to work on the hexactinellid reef (October 2007) **C** Henry with Dr Kim Conway, the geologist who discovered the Canadian hexactinellid reef, ibid; **D** Henry demonstrating sponge taxonomy techniques in a workshop at the 10^th^ World Sponge Conference, Galway, 25–30 June 2017. Images reproduced with permission.

Dr Henry Michael Reiswig of Saanichton, British Columbia, Canada (born 8 July 1936 in St. Paul, Minnesota, USA), died on 4 July 2020 at the age of 83, in his garage laboratory, doing what he loved most: science (Fig. 18). Henry was a marine biologist and globally renowned expert on the glass sponges (Hexactinellida), contributing enormously to knowledge of sponges in general.

Henry began his career at the University of California, Berkeley and completed a PhD at Yale University, after which he served as Professor of Biology at McGill University and Redpath Museum, Montreal, from 1972, until he officially retired in 2001. Those who knew him well chuckled at his ‘retirement’: in 2020 he was still hard at work describing the glass sponge fauna of New Zealand with his Kiwi and German friends. After formal retirement, he took up ‘post-retirement’ offices at the University of Victoria and the Royal British Columbia Museum in Victoria, B.C., ever busier and juggling numerous projects with colleagues and students all over the world.

Henry leaves an enormous legacy: his beloved wife, Ann, who died in February 2019 and their three wonderful daughters, Jennifer, Penelope, and Amy; more than 100 publications including journal articles, book chapters, and conference presentations; several sponge species and a sponge-derived secondary metabolite named after him, and hundreds of research colleagues and students who loved and respected him.

Henry also leaves a huge legacy at the National Institute of Water and Atmospheric Research (NIWA), New Zealand. He first began his work on New Zealand glass sponges in the 1980s as a visiting scientist at NIWA. Dr Dennis P. Gordon, now emeritus at NIWA, and then editor of the NIWA Biodiversity Memoir series, encouraged Michelle’s involvement and working with Henry, helping him make progress on this dauntingly huge project: to identify and name more than 329 glass sponges that had been collected by the original New Zealand Oceanographic Institute. With a sense of trepidation, Michelle began to work closely with Henry, ably assisted by the NIWA Invertebrate Collection manager, Sadie Mills. The current collection now includes more than 2000 glass sponges, most examined by Henry. Together we were able to get two major NIWA Biodiversity Memoirs, on the dictyonal and euplectellid glass sponges, over the line, and were in the process of describing the last two big groups, the Rossellidae and Amphidiscophora, when the shocking news arrived.

How do we go on? Well, continue in his name we do. Henry was utterly dedicated to his work and had a formidable intellect; for all his profound knowledge, he was also slightly ‘weird’ and wonderful, and there was nothing more fun than sitting with Henry, after a conference lecture, enjoying a cool beer in the sunshine. In the words of his official family obituary, he was, “a rascal, a scholar, a deeply moral man, and is profoundly and deeply missed.”

## Supplementary Material

XML Treatment for Bathydorus

XML Treatment for Nubes

XML Treatment for Scyphidium

XML Treatment for Caulophacus (Caulophacus)

XML Treatment for Rossellidae

XML Treatment for Rossellinae

XML Treatment for Bathydorus

XML Treatment for Bathydorus poculum

XML Treatment for Nubes

XML Treatment for Nubes tubulata

XML Treatment for Nubes poculiformis

XML Treatment for Vazella

XML Treatment for Scyphidium

XML Treatment for Scyphidium australiense

XML Treatment for Scyphidium variospinosum

XML Treatment for Lanuginellinae

XML Treatment for Caulophacus

XML Treatment for Caulophacus (Caulophacus)

XML Treatment for Caulophacus (Caulophacus) discohexaster

XML Treatment for Caulophacus (Caulophacus) serpens

XML Treatment for Caulophacus (Caulophacus) ramosus
